# Assigning the Low-Energy Excitons in Far-Red Photosystem II through Atomistic Modeling

**DOI:** 10.1021/acs.jpcb.6c05218

**Published:** 2026-10-21

**Authors:** Samuele Giannini, Luca Ancillotti, Lorenzo Cupellini

**Affiliations:** Dipartimento di Chimica e Chimica Industriale, 9310 Università di Pisa, Via G. Moruzzi 13, 56124 Pisa, Italy

## Abstract

Far-red light photoacclimation of cyanobacteria extends the spectral range of oxygenic photosynthesis through the incorporation of chlorophylls (Chls) *d* and *f* into natural photosystems, but the energetic organization of these pigments remains poorly understood. Here, we combine electronic-structure calculations with atomistic QM/MM and polarizable QM/MM approaches to determine the site energies, electronic couplings, and excitonic structure of the far-red PSII from *Synechococcus* sp. PCC 7335. In particular, we use benchmark calculations against spin-component-scaled CC2 to assess the accuracy of long-range corrected density functionals for describing the relative energetics of Chls *a*, *d*, and *f*. Our calculations reveal that the energetic tuning of the far-red pigments is governed primarily by protein electrostatics, whereas structural distortions and environmental polarization make comparatively minor contributions. We find significant excitonic interactions within the subset of CP47 chlorophylls, while the reaction-center Chl *d* remains largely localized. The resulting excitonic Hamiltonian quantitatively reproduces both the far-red absorption spectrum and the sequence of exciton energies extracted from two-dimensional electronic spectroscopy, enabling a first-principles assignment of the low-energy excitons to specific Chl *f* and Chl *d* pigments. These results provide an atomistic picture of the far-red excitonic landscape and establish a quantitative basis for future simulations of excitation energy transfer and charge separation in far-red photosystems.

## Graphical abstract

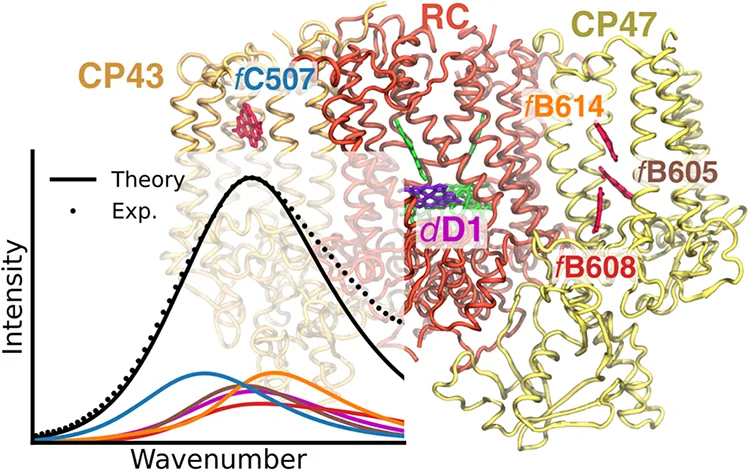

## 1 Introduction

Oxygenic photosynthesis converts solar energy into chemical energy through a series of highly efficient light-harvesting and charge-separation processes occurring within Photosystem I (PSI) and Photosystem II (PSII). In the photosystems of most photosynthetic organisms, these processes rely almost exclusively on chlorophyll (Chl) *a*, limiting efficient light harvesting to wavelengths below approximately 700 nm.^1,2^ Some cyanobacteria, however, have evolved the remarkable ability to exploit far-red light through a reversible acclimation process known as Far-Red Light Photoacclimation (FaRLiP),^3^ in which the photosynthetic apparatus is extensively remodeled to incorporate the red-shifted chlorophylls *d* and *f*.^3–7^ As a result, these organisms extend oxygenic photosynthesis to wavelengths approaching 750 nm while maintaining relatively high photochemical efficiencies.^8^

Operating under such low-energy illumination presents an important energetic challenge. Unlike Chl *a*, which carries methyl and vinyl substituents at positions C2 and C3 of the tetrapyrrole ring, respectively, Chl *d* contains a formyl group at C3, whereas Chl *f* carries a formyl group at C2 (See Figure S1). These substitutions shift the absorption of the pigments to the red, lowering the excitation energies of both the antenna and reaction-center chlorophylls and thereby reducing the thermodynamic driving force for excitation energy transfer and charge separation. Understanding how far-red photosystems preserve efficient photochemistry despite these constraints has therefore become a central question in photosynthesis research.^8,9^

Despite extensive experimental efforts, the energy landscape of far-red-adapted photosystems remains incompletely understood. Time-resolved spectroscopy experiments, including two-dimensional electronic spectroscopy (2DES), have revealed the existence of several low-energy exciton states associated with the far-red pigments. However, the complexity and spectral congestion of spectroscopic signals do not allow for resolving all energy levels.^10–13^ Hence, experimental data are often interpreted in terms of simplified “pigment compartments”, without a definitive assignment of excited states. Thus, the identity and role of the lowest-energy species remain uncertain.^10^

Atomistic quantum-mechanical (QM) calculations provide a natural route to bridge this gap. Structure-based simulations have been used to interpret spectroscopic signatures and suggest possible locations of low-energy sites in PSI.^5^ In some cases, however, these simulations rely on empirical assumptions about pigment–protein and pigment–pigment interactions, limiting their predictive power. Quantum mechanics/molecular mechanics (QM/MM) approaches can directly predict pigment site energies and electronic couplings from the protein structure while explicitly accounting for the electrostatic and polarization effects of the protein environment.^14,15^ Although similar methodologies have been successfully applied to canonical photosystems,^16–21^ their extension to far-red photosystems has only recently appeared.^17,22^ This has been hindered not only by the need for reliable electronic-structure methods capable of accurately describing Chl *d* and Chl *f*, but also by the limited availability of high-resolution structural models suitable for atomistic simulations.

In this respect, cryo-EM structures of far-red (FR) PSII from *Synechococcus* sp. PCC 7335 were characterized and the positions of one Chl *d* and four Chl *f* molecules were identified by Gisriel and co-workers, providing a good structural framework for atomistic investigations of the far-red complex.^23–25^ Even more recently, Leong et al.^26^ reported high-resolution cryo-EM structures of another far-red PSII *Chroococcidiopsis thermalis* PCC 7203 and *Calothrix* sp. NIES-3974. By combining structural, spectroscopic, and comparative analyses, they assigned the four Chl *f* binding sites and correlated them with the low-temperature absorption bands, thereby providing an experimental framework for interpreting the energetic organization of far-red PSII.^26^ Nevertheless, a quantitative, first-principles description of the excitonic landscape and a rigorous assessment of the role of protein electrostatics and excitonic interactions in determining these energy levels are still lacking. The central objective of this work is to address this gap, and establish a reliable atomistic description of the excitonic landscape of far-red PSII that connects its molecular structure to the experimentally observed low-energy excitons.

Here we present a benchmarked first-principles investigation of the excitonic landscape of the monomeric far-red PSII from *Synechococcus* sp. PCC 7335 (reported in Figure 1).^23^ We first assess the performance of several density functionals against spin-component-scaled CC2 calculations for isolated chlorophylls, allowing us to identify computational approaches capable of accurately reproducing the relative energetics of Chl *a*, Chl *d*, and Chl *f*. We then combine multiscale polarizable quantum mechanics-molecular mechanics (QM/MM) calculations of site energies and excitonic couplings to construct a fully atomistic excitonic Hamiltonian for the far-red pigments. The resulting model reproduces the experimental absorption spectra and the exciton energies extracted from 2DES experiments,^10^ enabling an assignment of the low-energy exciton states to specific Chl *f* and Chl *d* pigments. Beyond providing a molecular interpretation of the far-red absorption band, this work establishes a framework for future studies of excitation-energy transfer and charge separation in far-red photosystems.

**Figure 1:**
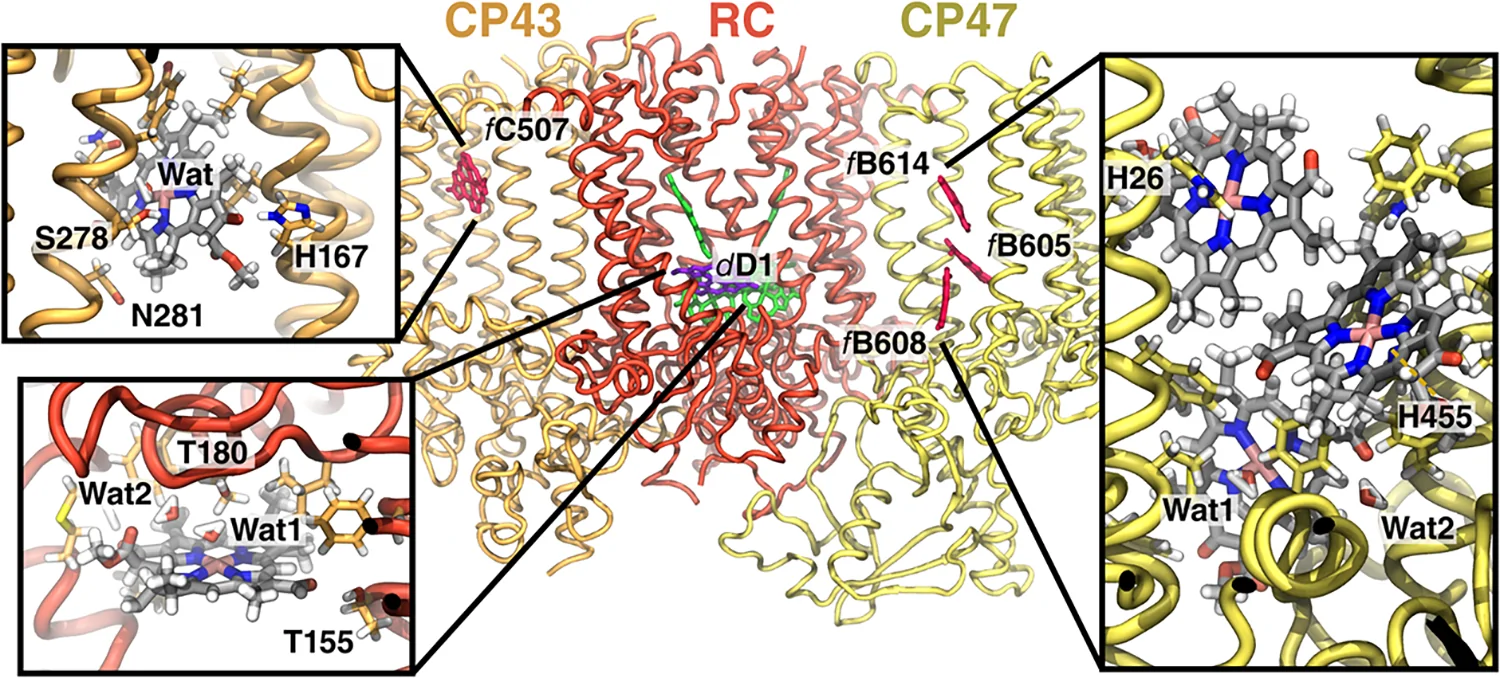
Structure of FR-PSII from *Synechococcus* sp. PCC 7335 (PDB: 7SA3)^23^ with the proposed sites for far-red Chls.

## 2 Methods

### 2.1 Structure Refinement and Optimization

We started our analysis by preparing the monomeric structure of the FR-PSII from *Synechococcus* sp. PCC 7335 as obtained from cryo-EM (PDB ID: 7SA3) in ref 23. The structure was refined using a combination of structural modeling, simulated annealing, and MM energy minimization following a protocol analogous to the one previously developed by some of us.^21^ In this way, we obtained the refined structure shown in Figure 1, where we have highlighted the four Chls *f* and the Chl *d* and their positions according to the assignment suggested in ref 23.

In more detail, the PDB structure was prepared using the tleap module of AmberTools,^27^ adding all missing heavy atoms, including those absent from the phytol tails of the Chls, and all hydrogen atoms. Steric clashes arising from the addition of a large number of missing heavy atoms were resolved by iteratively rotating the dihedral angles of the added segments using a Monte Carlo scheme.^21^ Plastoquinone-9 molecules were poorly resolved in the electron density map and were therefore removed from the model. Protein residues were assigned their standard protonation states, except for histidines coordinating Chl Mg ions, whose N_δ_- or N_ϵ_-protonation states were selected to preserve metal coordination.

The resulting model was first subjected to a constrained energy minimization in which only the newly added atoms and hydrogen atoms were allowed to move. This was followed by simulated annealing to allow the added atoms to relax, for a total of 300 ps using a 1 fs time step and the Andersen thermostat. This simulation was performed without periodic boundary conditions, using a nonbonded cutoff of 30 Å. Only the phytol tails and hydrogen atoms were able to move, while all other atoms were kept frozen to their initial positions. The system was heated from 100 to 600 K over 20 ps, maintained at 600 K for 80 ps, cooled from 600 to 300 K over 50 ps and from 300 to 100 K over a further 50 ps, and finally cooled from 100 to 0 K over 100 ps.

The structure obtained at the end of the annealing procedure was further refined through 5000 minimization cycles, comprising 2500 steps of steepest descent minimization followed by 2500 steps of conjugate gradient minimization. During this final refinement, the protein backbone and selected cofactor atoms were harmonically restrained to their reference positions using a force constant of 4.0 kcal mol^–1^ Å^–2^. For Chls, only the chlorin ring was restrained. Protein-backbone atoms belonging to Mg-coordinating residues were excluded from these restraints, allowing the coordinating residues and the remaining unrestrained atoms to relax. In short, restraints were applied to the structural features with the highest confidence, whereas the protein side chains were left free to move. This procedure yielded the final optimized structural model used in the subsequent calculations. The refinement protocol corrects structural inaccuracies arising from the limited resolution of the cryo-EM structure and prevents steric clashes from influencing the structure during the minimization.

All structural refinements were performed using AmberTools.^27^ The ff14SB force field was used to describe the protein.^28^ Parameters for chlorophylls, pheophytins, heme groups, and the oxygen-evolving complex were taken from previous studies.^29^ Lipid parameters were derived from the Lipid21 Amber force field.^30^ Parameters for Chl *f* and Chl *d* were generated by optimizing the structure at the B3LYP/6–31G­(d) level of theory and obtaining RESP charges at the B3LYP/6–311G­(d,p) level.

After refinement, to perform subsequent electronic structure calculations, the ground state geometry of each Chl type (i.e., the four Chl *f* and one Chl *d*) was optimized at QM/MM level of theory within the ONIOM scheme, as implemented in Gaussian 16.^31^ The Chls macrocycle, including all atoms up to the phytol, and the axially coordinated side chains were included in the QM region. The remaining atoms of the phytyl chain were assigned to the MM region and kept frozen during geometry optimization. The QM region was described using DFT at the B3LYP/6–31G­(d) level with empirical dispersion,^32^ whereas the MM region was described using the same force field as for the previous refinement. This optimization strategy has proven effective for far-red absorbing LHCs containing only Chl *a*.^33^ All the following calculations were performed on this optimized structure.

### 2.2 Exciton Hamiltonian and Optical Spectra

The excited states of the far-red pigments were described within the exciton model, considering the Chl *f* and *d* of the FR-PSII systems optimized at QM/MM level as previously described. The exciton Hamiltonian was constructed as

1 $${Ĥ}_{\mathrm{el}}=\sum_{i}\epsilon_{i}\left|i\right\rangle \left\langle i\right|+\sum_{i\neq j}V_{\mathrm{ij}}\left|i\right\rangle \left\langle j\right|$$

where ϵ_*i*_ is the site energy of pigment *i* and *V*_*ij*_ is the excitonic coupling between pigments *i* and *j*. For the purpose of our analysis, we considered only a single excitation (i.e., the lowest-energy Q_*y*_ excitations) per site. Diagonalization of *Ĥ_el_* yields the exciton states

2 $$\left|a\right\rangle =\sum_{i}c_{\mathrm{ia}}\left|i\right\rangle$$

with exciton energies *E*_*a*_ and coefficients *c*_*ia*_ describing the contribution of pigment *i* to exciton *a*.

Site energies were computed using either an electrostatic embedding QM/MM or a polarizable QM/MM (QM/MMPol) approach,^34–36^ in which the environment is allowed to polarize in response to the QM density, in turn creating a field that acts back on the QM part. The presence of the polarizable embedding allows for describing the effect of the atomistic environment of the protein on the site energies and transition densities of the pigments. For all QM/MM and QM/MMPol calculations, the QM region comprised the chlorophyll (or pheophytin) macrocycle. In contrast, the phytyl chain, surrounding cofactors, and protein residues within 30 Å were treated at the MM level. In QM/MMPol calculations, MM atoms within 15 Å of the QM region were considered polarizable.

As done in Section 3.2, to analyze the contributions of various residues to the electrostatic shift on the Q_*y*_ transitions, we employed the charge-density coupling (CDC) scheme.^37–39^ Briefly, we estimated the electrochromic shift (QM/MM *minus* vacuum) as the Coulomb interaction between difference atomic charges (Δ*q*_*i*_ = *q*_*i*_^(*S*_1_)^–*q*_*i*_^(*S*_0_)^) and the MM charges. The difference charges Δ*q*_*i*_ were determined by a fitting of the electrostatic potential (ESP) generated by the difference electronic density (*S*_1_–*S*_0_). The difference ESP for the Chls are shown in Figure S2.

Excitonic couplings were calculated using the Coulomb interaction between the transition densities computed at QM level as described in eq 3, explicitly taking into account the effect of the polarizable environment.^34,40^ Specifically, within the MMPol framework, the coupling is expressed as

3 $$V_{\mathrm{ij}}=\iint \frac{\rho_{i}^{\mathrm{tr}}\left(r\right)\rho_{j}^{\mathrm{tr}}\left(r^{\prime }\right)}{\left|r-r^{\prime }\right|}drdr^{\prime }-\sum_{k}\left(\int \frac{r_{k}-r}{|r_{k}-r{|}^{3}}\rho_{i}^{\mathrm{tr}}\left(r\right)dr\right)\cdot \mu_{k}^{\mathrm{ind}}\left(\rho_{j}^{\mathrm{tr}}\right)$$

where ρ_*i*_^tr^ and ρ_*j*_^tr^ are the transition densities of pigments *i* and *j*, respectively. The summation runs over all polarizable atoms of the environment, and **μ**_*k*_^ind^(ρ_*j*_^tr^) is the dipole induced on atom *k* by the electric field generated by the transition density of pigment *j*. The first and second terms of eq 3 represent the bare Coulomb coupling, *V* ^Coul^, and the explicit environment-mediated contribution in the MMPol formulation, *V* ^MMPol^, respectively.

Exciton–phonon coupling was described through spectral densities computed for each Chl obtained starting from the optimized structure and using the vertical gradient approach to get the vibronic coupling as described in Section S3 of the SI. Static inhomogeneous disorder was incorporated by generating 1000 realizations of the excitonic Hamiltonian, in which site energies were independently sampled from a Gaussian distribution with a standard deviation of 25 cm^–1^ for the comparison of the experimental spectra at 300 K. This commonly used approximation neglects possible correlations between the static site-energy fluctuations of different pigments. We note that in this regime the spectrum is insensitive to the value of the standard deviation (Figure S14). For each realization of the disorder, the absorption spectrum was calculated using the full cumulant expansion (FCE) approach.^41^ This theory is based on the second-order cumulant expansion and includes nonsecular and non-Markov effects. Contributions of individual pigments to the absorption line shape were extracted as described in ref 42.

### 2.3 Excited-State Calculations

For each chromophore, *i*, excited-state calculations were performed using time-dependent density functional theory (TD-DFT) at different levels of theory, considering hybrid-GGA, meta-hybrid GGA and long-range corrected hybrids (see Section S6). As discussed in the Results, unless otherwise stated, for the calculation of the exciton Hamiltonian, we used the best-performing functionals: M06–2X and ωB97X-D*, with the 6–31G­(d) basis set. All TD-DFT and DFT calculations were performed with a locally modified version of Gaussian 16.^31^ The asterisk denotes that the long-range separation parameter in ωB97X-D was set to ω = 0.3 to get a functional consistent with ωB97X-V,^43^ which was adopted for photosystems in ref 44. We note that there remains a difference between these functionals concerning the dispersion correction, which, however, does not influence excited states.

Higher-level reference calculations were performed using SOS-CC2 and SCS-CC2 methods^45–47^ using def2-TZVP basis-set as implemented in TURBOMOLE V7.5.1.^48^ Spin-corrected versions of CC2 were employed as reference, as standard CC2 was found to be insufficiently accurate for *Q*_*y*_ electrostatic shifts.^49^ These calculations were performed within the electrostatic embedding QM/MM framework, using the same charges as in QM/MM TD-DFT calculations.

## 3 Results

This section is organized as follows. We first assess the performance of the DFT functionals in describing the differences among isolated Chls *a*, *d*, and *f*. We then analyze the site energies of red-shifted Chls in FR-PSII and disentangle geometrical, electrostatic, and polarization effects. Next, we construct the exciton Hamiltonian of FR-PSII and use it to interpret the absorption spectrum and assign the experimentally observed low-energy excitons. Finally, we discuss the reliability of the proposed Chl *f* site assignments.

### 3.1 Benchmarking the Excitation Energies of Far-Red Chlorophylls

We begin our analysis by benchmarking the Q_*y*_ excitation energies of Chls *a*, *d*, and *f*. To this end, we performed TD-DFT calculations employing several exchange-correlation functionals in both the gas phase and methanol, the latter described through the polarizable continuum model (PCM).^50^ The TD-DFT results in gas phase were compared against higher-level SOS-CC2 and SCS-CC2 calculations (see Section 2.3 for computational details).

The calculated excitation energies are reported in Table 1 for both methanol and gas-phase conditions, respectively. All calculations in this table were performed using the same starting geometries, obtained by optimizing each chlorophyll at the B3LYP/6–31G­(d) level with methanol described using PCM. All methods correctly predict a red shift of both Chls *d* and *f* relative to Chl *a*. The magnitude of the shift, however, varies with the level of theory. In particular, B3LYP and M06–2X reproduce the relative ordering of the pigments but systematically overestimate the absolute excitation energies. In contrast, ωB97X-D* provides lower excitation energies, but predicts nearly degenerate Chl *d* and Chl *f* transitions. The SOS-CC2 and SCS-CC2 calculations *in vacuo* reproduce the experimental trend more closely, yielding Chl *a*–Chl *d* and Chl *a*–Chl *f* energy separations of approximately 600–1000 cm^–1^. Although the PCM solvent was not included here, the Chl *d*– Chl *f* difference is not expected to vary significantly in the solvent, at least according to the TD-DFT calculations. We refer to Table S2 for other functionals.

**Table 1:** Q_*y*_ Vertical Absorption Energies Calculated with TD-DFT/6-31G­(d) and Different Functionals^a^,^b^

	Chl *a*	Chl *d*	Chl *f*	Δ*ν̃*_*a–f*_	Δ*ν̃*_*a–d*_	Δ*ν̃*_*d–f*_
In vacuo						
B3LYP	17328	16677	16544	784	651	133
M06–2X	17462	16721	16676	786	741	45
ωB97X-D*	16612	15695	15746	866	917	–51
SOS-CC2	16618	15901	15640	978	717	261
SCS-CC2	16895	16297	16049	846	597	248
Methanol (PCM)						
B3LYP	16990	16167	16022	968	823	145
M06–2X	17175	16293	16189	986	882	104
ωB97X-D*	16385	15337	15313	1072	1048	24
Exp.^b^	15053	14362	14124	929	691	238

a Experimental values in methanol solvent from refs 2,51 .

b The *in vacuo* results are compared with spin-corrected CC2/def2-TZVP, whereas methanol results were obtained using PCM. All values are in cm^–1^.

A key result is that Chl *d* and Chl *f* remain energetically very close, with calculated separations ranging from a few tens to a few hundred cm^–1^ depending on the electronic structure method. This small energy difference highlights the challenge of assigning their respective spectroscopic signatures inside the photosystems. Overall, our benchmark indicates that the sizable red shift relative to Chl *a* can be reasonably predicted with TD-DFT methods, while accurately resolving the relative ordering of Chl *d* and Chl *f* requires a high-level treatment. In fact, M06–2X yields a too small difference between Chl *d* and Chl *f*, and ωB97X-D* even predicts the wrong order in gas phase with respect to the CC2 references. The error made in capturing the position of Chl *d* with respect to Chl *f* by the three functionals B3LYP, M06–2X and ωB97X-D* compared to SCS-CC2 for the isolated pigments are 115, 203, and 299 cm^–1^.

### 3.2 Electrostatic Contribution to Chlorophyll Site Energies

We now investigate the excitation energies of the five red-shifted chlorophylls in FR-PSII within their native protein environment to disentangle the respective contributions of pigment geometry and protein electrostatics to energy tuning. To this end, we compare the excitation energies of the pigments embedded in FR-PSII with those of the corresponding isolated chlorophylls calculated in *vacuo*. We further compare the TD-DFT calculations with the SCS-CC2 reference.

Figure 2a shows the change in the Q_*y*_ energy from the isolated pigment (Chl *d* or *f*) to FR-PSII, calculated with and without the environment. Geometric effects alone produce relatively small spectral shifts, generally below 100 cm^–1^, which indicates minor effects from structural deformations of the Chls. In contrast, including the protein environment at the QM/MM level induces red shifts of up to ∼500 cm^–1^, showing that electrostatic interactions have a primary role in tuning pigment energies. This result is in line with the model used for far-red PSI in ref 5, where the protein effects on pigments have been determined from purely electrostatic calculations. The largest effects are observed for *f*B614, *f*C507, and the reaction-center pigment *d*D1. Importantly, the trends predicted by TD-DFT with either ωB97X-D* or M06–2X closely follow the SCS-CC2 reference data (Figure 2b), which provides confidence that the TD-DFT methods capture the essential features of the protein-induced electrochromic spectral tuning. In contrast, the B3LYP functional (Figure S7) very poorly describes the electrostatic effects of the protein environment, predicting similar shifts for all pigments. We refer to Figure S8 for the results obtained with other DFT functionals. These results indicate that the far-red site energy ladder of FR-PSII arises primarily from environmental modulation of selected Chl *f* and Chl *d* pigments rather than from intrinsic differences in pigment geometry.

**Figure 2:**
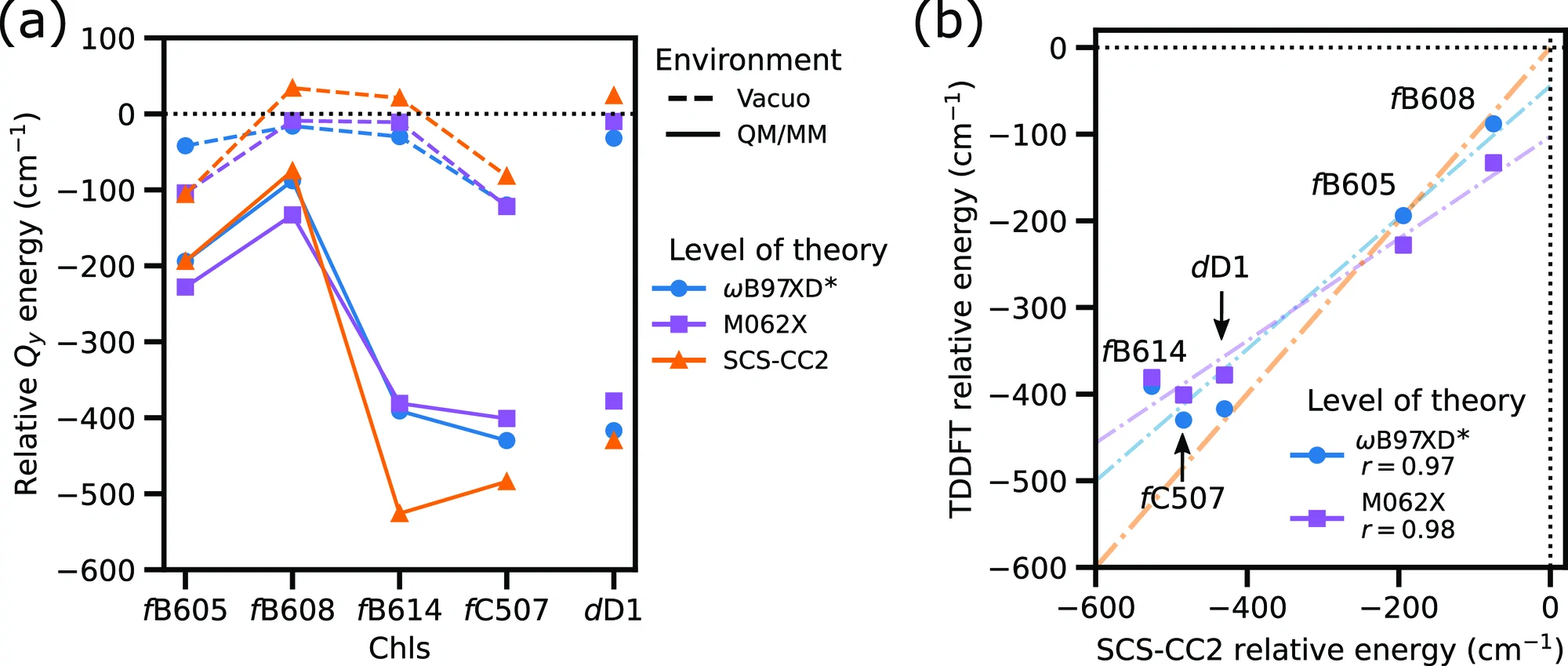
(a) Changes in the Q_*y*_ excitation energies of the four Chl *f* pigments (*f*B605, *f*B608, *f*B614, and *f*C507) and the reaction-center Chl *d* (*d*D1), relative to the excitation energies of the isolated pigments optimized in vacuo (horizontal dotted line). Excitation energies were calculated at the optimized QM/MM geometry as described in Section 2.1. Dashed lines correspond to excitation energies calculated *in vacuo* on the optimized QM/MM structure, thereby accounting only for the effect of geometrical distortions. Solid lines correspond to QM/MM excitation energies computed for the same geometry, thus including the electrostatic effect of the protein environment. Different colors denote the various electronic structure methods. (b) Correlation between protein-induced shifts predicted at QM/MM level by TD-DFT and SCS-CC2 for the Chl *f* pigments. Both functionals reproduce the relative ordering and magnitude of the protein-induced spectral tuning.

To rationalize these findings and understand which residues play a more prominent role in the energy stabilization, we simplified the electrostatic contributions employing the charge-density coupling (CDC) scheme.^37–39^ Despite its simplicity, this scheme yields results in semiquantitative agreement with the QM/MM calculations (Table S6 in the SI), and recovers the large electrochromic red shifts of Chls *f*C507 and *f*B614. Therefore, we can employ these calculations to compute the contribution of each amino acid or cofactor to the electrochromic shift, shown in Figure 3 for these Chls (see Figure S10 for the other Chls). We stress that the contributions obtained from the CDC analysis should be interpreted with some caution, as CDC represents a first-order estimation of the electrostatic effect. Nonetheless, the contributions estimated with CDC correlate well with those obtained from QM/MM calculations (Figure S11).

**Figure 3:**
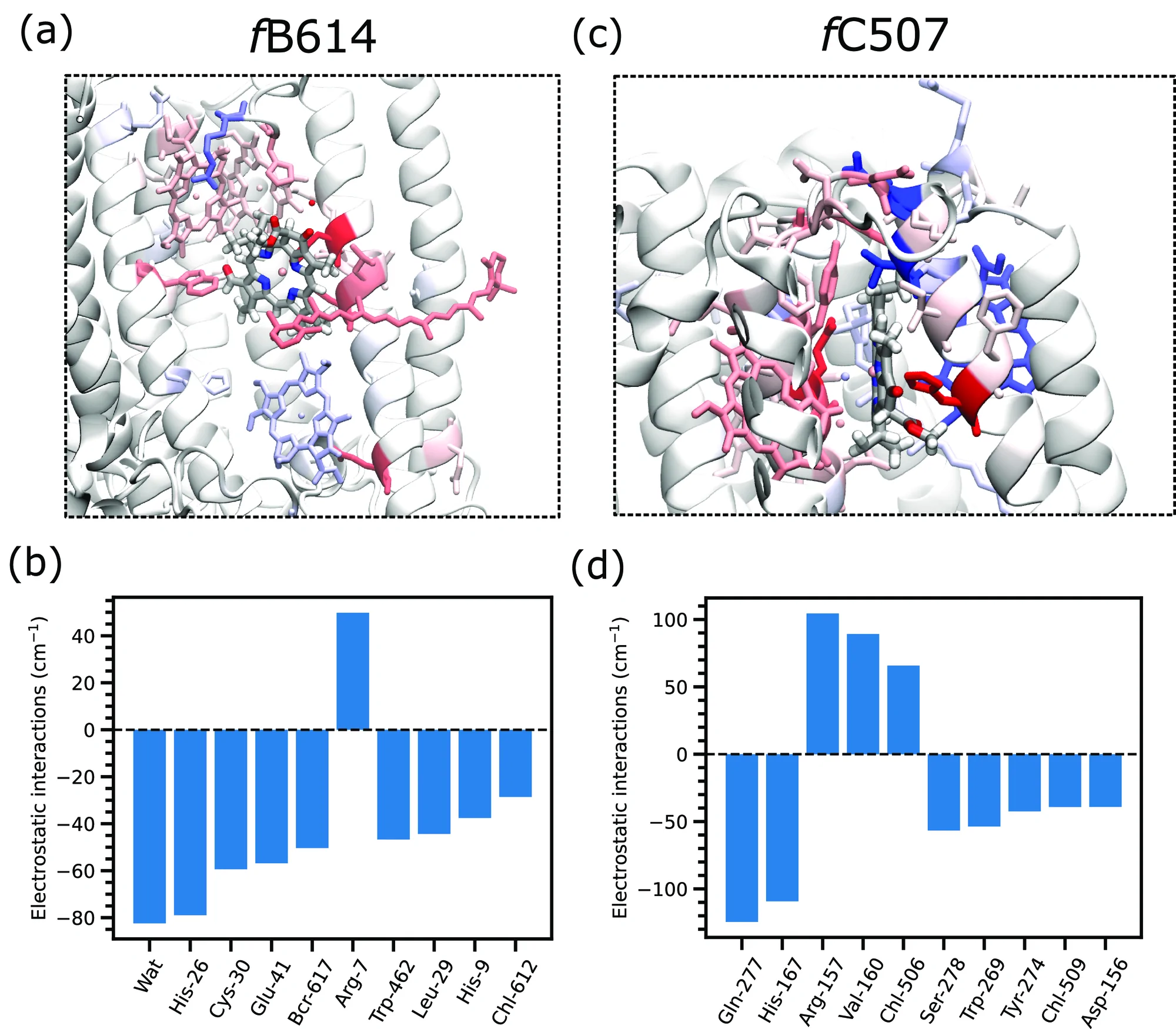
Electrostatic contributions to the electrochromic shifts of Chl fB614 and Chl fC507. Panels (a) and (c) show the local protein environment surrounding Chl fB614 and Chl fC507, respectively, with residues colored according to the sign of their electrostatic contribution (red: negative; blue: positive). Panels (b) and (d) report the corresponding decomposition of the electrochromic shift into the individual contributions from the surrounding residues and cofactors. For clarity, the phytol tails of the chlorophylls have been omitted.

For Chl *f*B614 Mg-binding His26 gives the largest red shift, together with a water molecule located close to the 13^1^ carbonyl. On the contrary, Chl *f*C507 is bound by a water molecule, which however results in a slight blue shift. Instead, the largest negative contributions are given by Gln277, at the same side of the ligating water, and by His167, which makes a H-bond with the 13^1^ carbonyl. Chl *d*D1 is mainly red-shifted by the H-bonded Thr155, as well as the special pair Chl P_*D1*_.

The relatively large electrostatic effect observed for two of the Chls *f* and for Chl *d*D1 suggests that Chls *d* and *f* have larger intrinsic sensitivity to electric fields relative to Chl *a*. Analogously, Maity et al. observed large electrostatic effects in Chls *c1* and *c2*.^52^ In particular, they showed that excitation energies of Chls *c1* and *c2* were more sensitive to directional homogeneous electric fields than that of Chl *a*. To understand the origin of the electrochromic shifts in FR-PSII, we applied the same analysis for Chls *a*, *d*, and *f* described at the ωB97X-D* level. Table S3 reports the excitation energy shifts obtained by applying homogeneous electric fields of 0.01 *a.u.* in the different Cartesian directions, as in ref 52. Surprisingly, homogeneous electric fields result in smaller red shifts for Chls *d* or *f* than for Chl *a*, or even in blue shifts. Furthermore, the observed red shifts are at most ∼140 cm^–1^, much smaller than the ones observed in the protein (see Figure 2). This unexpected result can be explained by noting that the density difference of Chls *d* and *f* is essentially quadrupolar, as can be deduced from the difference ESP in Figure S2. As such, these Chls are much more sensitive to *inhomogeneous* electric fields, such as the ones found in FR-PSII.

### 3.3 Polarization Contribution to Chlorophyll Site Energies

In Figure 4, we assess the effect of environmental polarization by comparing standard QM/MM with QM/MMPol calculations, in which the MM environment is allowed to respond self-consistently to the electronic excitation of the chlorophyll pigments (see Methods for details). The inclusion of polarization has only a minor impact on the relative energetic ordering of the pigments, indicating that the site-energy pattern is primarily determined by the static electrostatic environment. Instead, polarization induces an approximately uniform stabilization of the excited states, resulting in an overall red shift of all pigments relative to the average energy of the Chl *f* manifold. Thus, explicit polarization effects contribute mainly to the absolute excitation energies, while leaving the relative energy landscape largely unchanged. Nevertheless, properly accounting for both electrostatic and polarization effects is needed to correctly capture the relative ordering of the various pigments.

**Figure 4:**
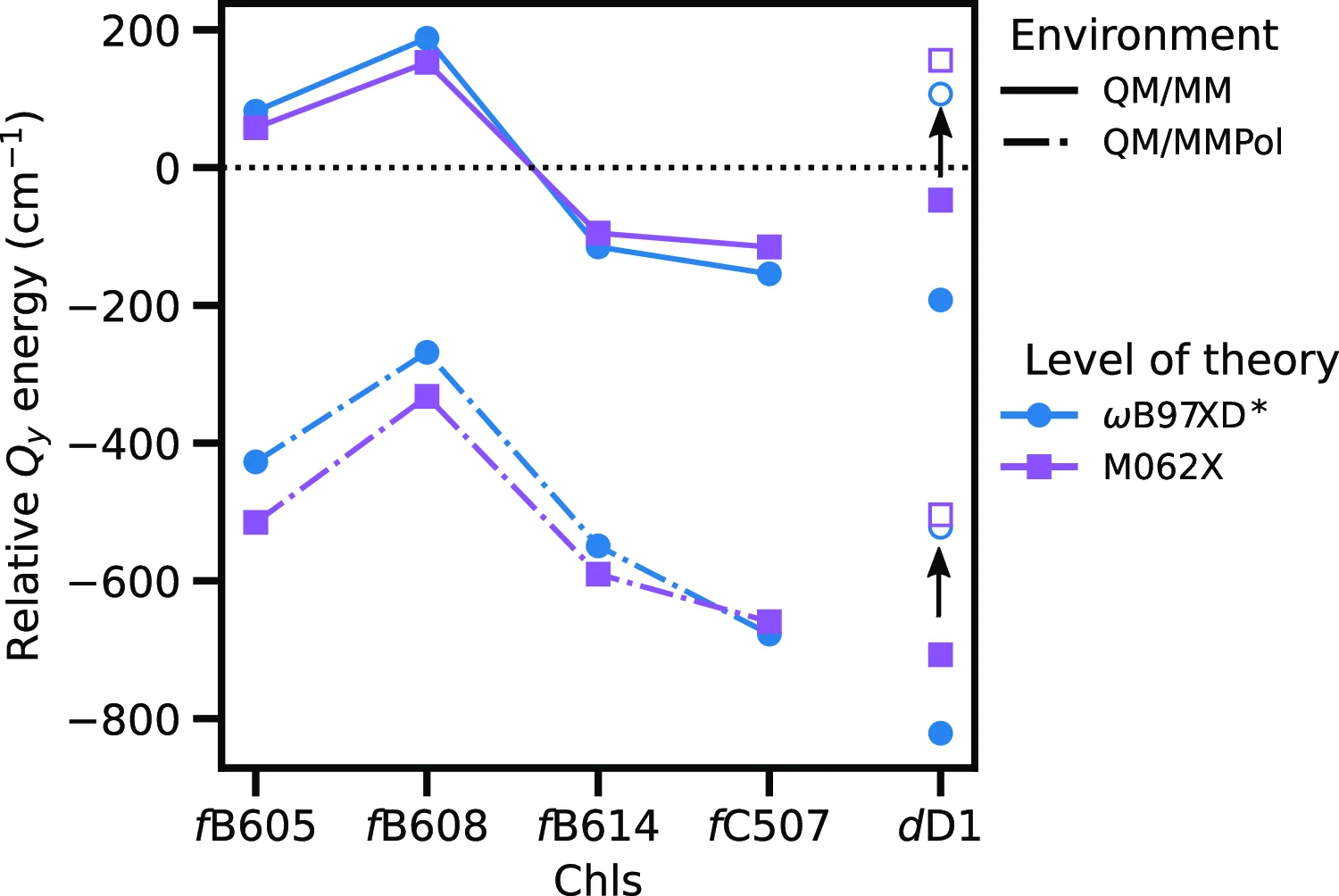
Relative Q_*y*_ excitation energies of the four Chl f pigments and the reaction-center Chl *d* (*d*D1) computed using standard QM/MM (solid lines) and polarizable QM/MMPol (dash-dotted lines) embedding at the same optimized QM/MM geometry. Results are shown for tuned ωB97X-D* and M06–2X. The zero of the energy scale is defined as the mean excitation energy of the Chls *f* to facilitate comparison between functionals. Open symbols for *d*D1 indicate excitation energies shifted to correct for the systematic deviation of the respective functionals relative to the SCS-CC2 reference values obtained for the isolated Chls as discussed in the text.

As discussed in Section 3.1, both ωB97X-D* and M06–2X exhibit different systematic errors in reproducing the absolute excitation energies of Chl *d* and Chl *f*, resulting in a systematic error on the *d*–*f* difference as compared to post-HF as well as experimental estimates. With this error, the energy of Chl *d*D1 would become similar or even lower than the red-most Chls *f*. To compensate for these functional-dependent offsets, constant shifts of 299 cm^–1^ and 203 cm^–1^ as indicated by empty symbols in Figure 4 were applied to the excitation energies obtained with ωB97X-D* and M06–2X, respectively. This correction changes the relative energetic ordering of the chromophores toward the correct experimental direction, as we discuss below in more detail.

We remark that our results are based on the static optimized structural model considered here. Within this limit, the protein electrostatic environment provides the dominant contribution to the energetic tuning of the far-red chlorophylls. Temperature-induced fluctuations tend to average out both electrostatic and geometric effects,^14^ therefore introducing a treatment of protein dynamics, for example with MD simulations, may affect the overall magnitude of the two effects.

### 3.4 Excitonic Effects on the Far-Red PSII

Having calculated and benchmarked the site energies of the different Chl *f* pigments, we next turn to the description of excitonic effects by constructing the Hamiltonian in eq 1. To this end, the excitonic couplings were computed from the interaction between the transition densities of the different Chls in the explicit protein environment using eq 3, as described in the Methods section. The resulting couplings (Table 2) indicate that the Chl *f* pigments in CP47 are appreciably coupled, reaching values as high as ≈116 cm^–1^ for the closely stacked *f*B605-*f*B608 pair (Mg–Mg distance of 11.4 Å) and −50 cm^–1^ for the *f*B605-*f*B614 (Mg–Mg distance of 10.2 Å).

**Table 2:** Excitonic Couplings (cm^–1^) Obtained Including the Effect of the Polarizable Environment for the Investigated Functionals with 6-31G­(d) Basis Set

Chl 1	Chl 2	Dist. Mg–Mg (Å)	M06–2X	ωB97X-D*
*d*D1	*f*B605	37.1	0.2	0.2
*d*D1	*f*B608	34.5	–2.2	–2.0
*d*D1	*f*B614	37.3	–2.8	–2.7
*d*D1	*f*C507	40.0	5.3	5.1
*f*B605	*f*B608	11.4	133.7	115.5
*f*B605	*f*B614	10.2	–55.4	–50.1
*f*B605	*f*C507	73.2	0.4	0.3
*f*B608	*f*B614	14.3	61.4	58.6
*f*B608	*f*C507	72.6	–0.3	–0.3
*f*B614	*f*C507	71.7	–0.4	–0.3

In contrast, *f*C507 in CP43 is more isolated and only weakly coupled to the reaction-center Chl *d*D1. This agrees with expectations from dipole–dipole interactions, which scale as *r* ^–3^, where *r* is the distance between the molecules.^53^ Although the couplings involving *d*D1 are comparatively small (smaller than 6 cm^–1^), they are sufficient to provide a pathway for energy transfer toward the RC. This is confirmed by the subnanosecond EET rates computed with generalized Förster theory (See Section S10 of the SI). In particular, we estimate EET times of ∼64 ps and ∼370 ps, respectively, from *f*C507 and from the CP47 domain. Notably, *f*C507 is the Chl *f* with the largest coupling to Chl *d*D1. The larger coupling and faster EET rate from the CP43 side are consistent with earlier estimates^54^ and with a faster excitation energy transfer from the CP43 side to the RC.

The effect of excitonic interactions on the energetic of the states is illustrated in Figure 5. Comparison of the site and exciton energies shows that excitonic mixing is primarily confined to the CP47 pigments *f*B605 and *f*B614, whose relatively small energy gap and sizable electronic coupling lead to the formation of two delocalized exciton states. In contrast, the weakly coupled pigments *d*D1 and *f*C507 retain a similar energy and an essentially localized character due to the weak excitonic interactions with the other pigments. Similar excitonic coupling patterns and energy ordering are obtained with both exchange-correlation functionals used, as already suggested for Chls *a*.^55^ This indicates that our computed assignment of the low-energy exciton is robust, and we discuss this in more detail below.

**Figure 5:**
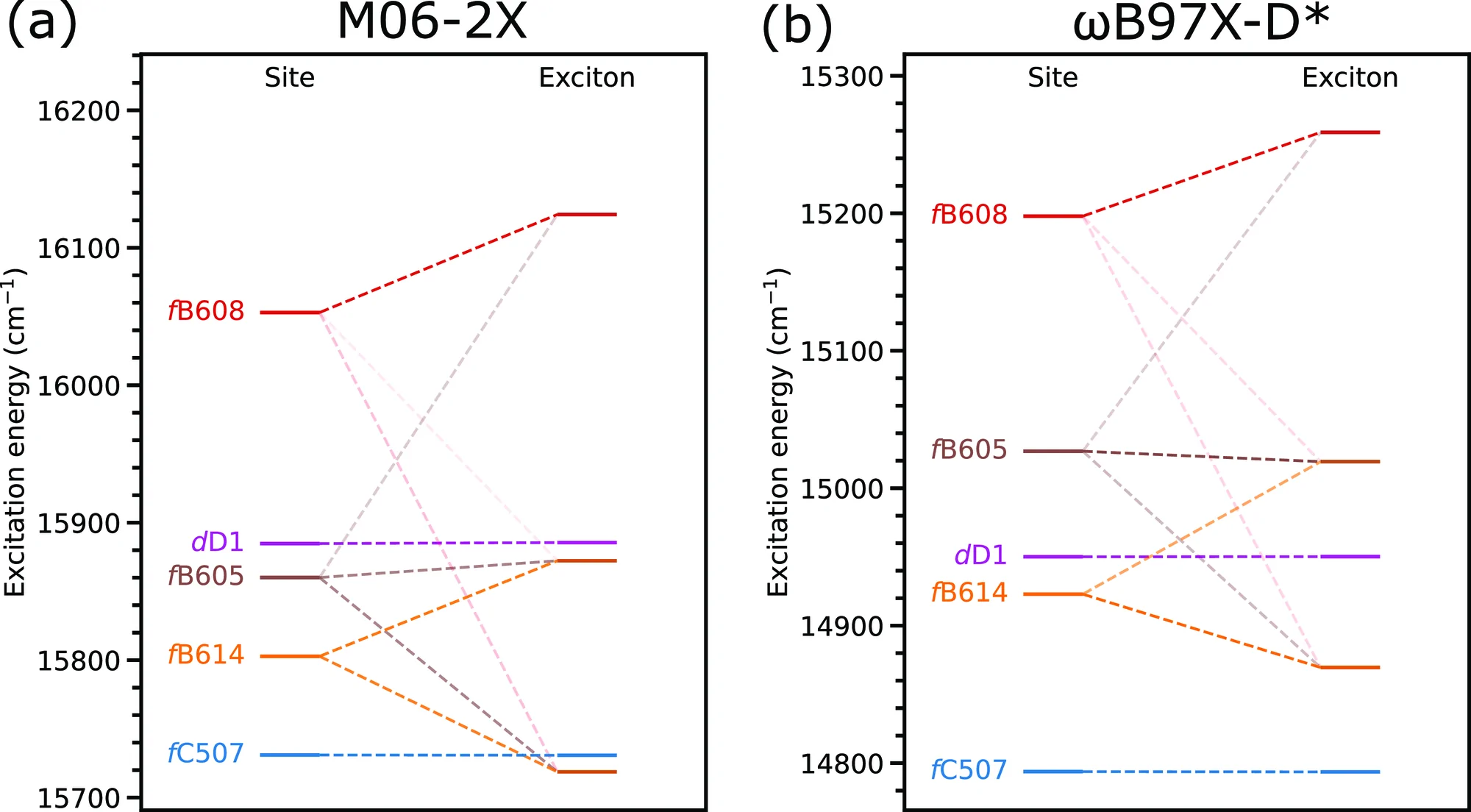
Energy levels of the investigated Chls computed using QM/MMPol excitonic couplings and excitation energies to compute the exciton Hamiltonian in eq 1. The excitation energies of individual Chls (sites) computed using (a) M06–2X or (b) ωB97X-D* are presented along with the corresponding excitonic levels. The dashed lines represent the contribution of the sites to the excitons; the opacity of the lines is proportional to the magnitude of the contribution. Strongly interacting Chl clusters are represented using the same color. The energy of the Chl *d*D1 has been adjusted as discussed in Figure 4.

We finally mention that the dipole strength of the CP47 domains is redistributed toward the higher excitons, while the lowest exciton is almost dark. This arises from the relative arrangement of *f*B605 and *f*B614, which results in a parallel orientation of their dipoles (see Figure S12 and Table S7).

### 3.5 Assignment of the Chlorophyll Contributions to the Low-Energy Absorption Band

In order to discuss the contribution of the different Chls *f* to the low-energy absorption band of this far-red PSII, we computed the steady-state absorption spectrum using the excitonic Hamiltonian in eq 1 and the full cumulant expansion approach.^41,42^ The simulated spectra are shown in Figure 6 for both the M06–2X and ωB97X-D* functionals (see Methods for details) and compared to the experimental room-temperature absorption measured in ref 23. In both cases, the calculated spectra, obtained using the individual spectral densities of the various Chls and applying global shifts of 1800 cm^–1^ and 930 cm^–1^, for M06–2X and ωB97X-D* functionals, respectively, to match the highest experimental peak, reproduce the experimental absorption band^23^ reasonably well, including the bandwidth. This indicates that the assignment of the Chls and the computed site energies and excitonic couplings provide a consistent description of the red-shifted absorption band. The intensity on the high-energy side of the spectrum is underestimated, leading to a less pronounced shoulder than observed experimentally. This shoulder may arise from the red-most tail of the Chl *a* band, which is not modeled here. Both exchange–correlation functionals predict essentially the same spectral features. Nevertheless, the best agreement is obtained with the ωB97X-D* functional. We note again that while the investigated TDDFT functionals reproduce reasonably well the relative excitation energies of the different pigments and the overall spectral line shape, the absolute excitation energies require a global shift to match the experimental spectrum as mentioned before.

**Figure 6:**
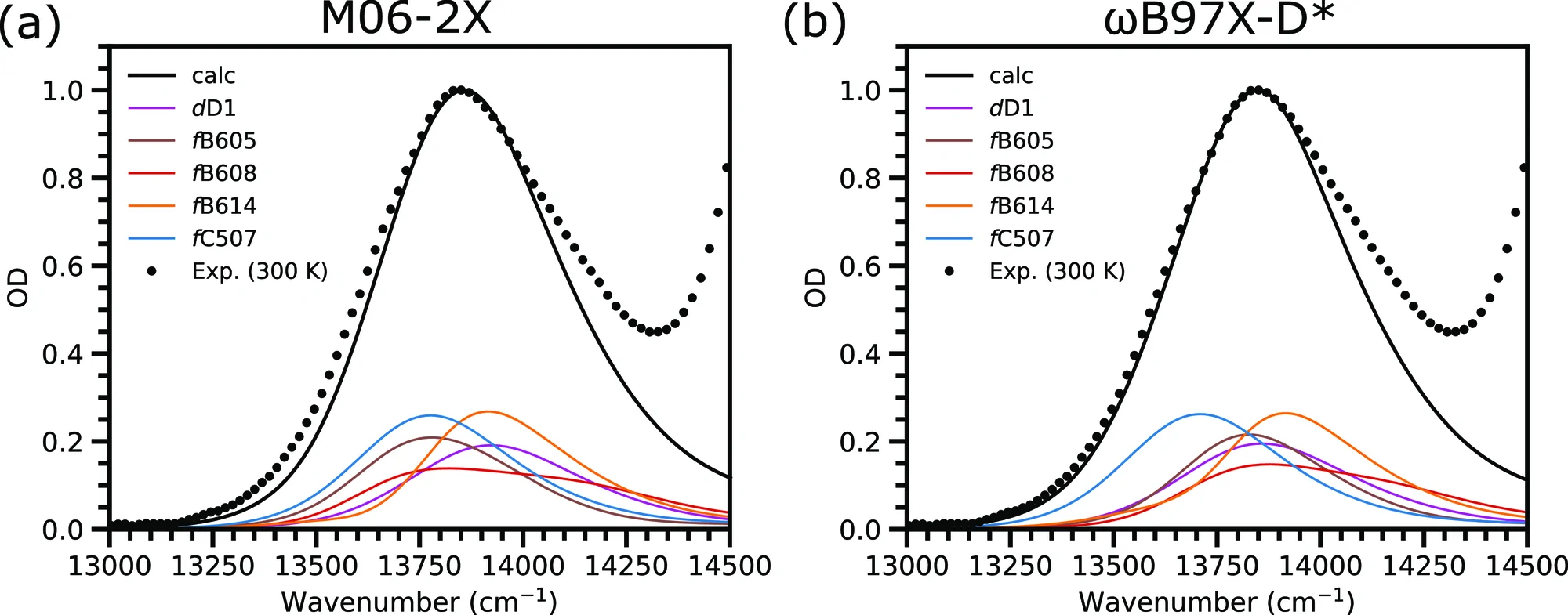
Calculated absorption spectra of the Chl *d*–*f* aggregate using the full cumulant expansion as explained in the text and the exciton Hamiltonian constructed with site energies and electronic couplings computed at the (a) M06–2X and (b) ωB97X-D* levels of theory. The black solid line shows the total calculated spectrum, while the black dots correspond to the experimental absorption spectrum at 300 K taken from ref 23. Colored curves represent the site-projected contributions of the individual pigments (*d*D1, *f*B605, *f*B608, *f*B614, and *f*C507) to the total spectrum. The spectra obtained with TD-DFT were globally shifted by 1800 cm^–1^ and 930 cm^–1^, for M06–2X and ωB97X-D* functionals, respectively, in order to match the highest experimental peak.

To understand the composition of the far-red band, we decomposed the spectrum into contributions from individual pigments. This reveals that the maximum of this band does not correspond to a single pigment but rather to contributions from all red Chls. The lowest-energy tail is mainly associated with the isolated *f*C507 located in CP43, whereas *f*B614 and *f*B608 contribute predominantly to the high-energy wing of the spectrum, despite the low site energy of *f*B614. In fact, the excitonic coupling between *f*B605 and *f*B614 and the dipole arrangement result in a redistribution of oscillator strength over the central part of the band, shifting the pigment contributions toward the blue. Nonetheless, switching off the excitonic interactions does not strongly change the overall line shape, and it does not lead to a worse agreement with the experiment (see Figure S13). Therefore, we cannot use the experimental steady-state absorption spectrum to validate the magnitudes of the individual excitonic couplings or the extent of excitonic mixing.

Remarkably, our calculated exciton energies closely reproduce the experimental trend of the energy levels identified by fitting the 77 K two-dimensional electronic spectroscopy (2DES) measurements of the same far-red PSII reported in ref 10. As shown in Figure 7, although our calculated vertical excitation energies are systematically higher by approximately 300 cm^–1^ than the energies extracted from the 2DES analysis, the two sets of values exhibit an almost perfectly parallel trend. This systematic offset is expected because the experimental energies also include vibrational relaxation of the exciton states (see Figure S3), which we do take into account in the spectra calculation. The excellent agreement in the relative energy spacing enables us to assign, within the accuracy of our protocol, the different experimental peaks to excitations predominantly localized on Chl *f* and Chl *d* pigments. In particular, our calculations place the Chl *d* excitation in the middle of the red-shifted exciton manifold, consistent with the assignment proposed on the basis of other spectroscopic experiments (see also Table S8).^10,25,56^

**Figure 7:**
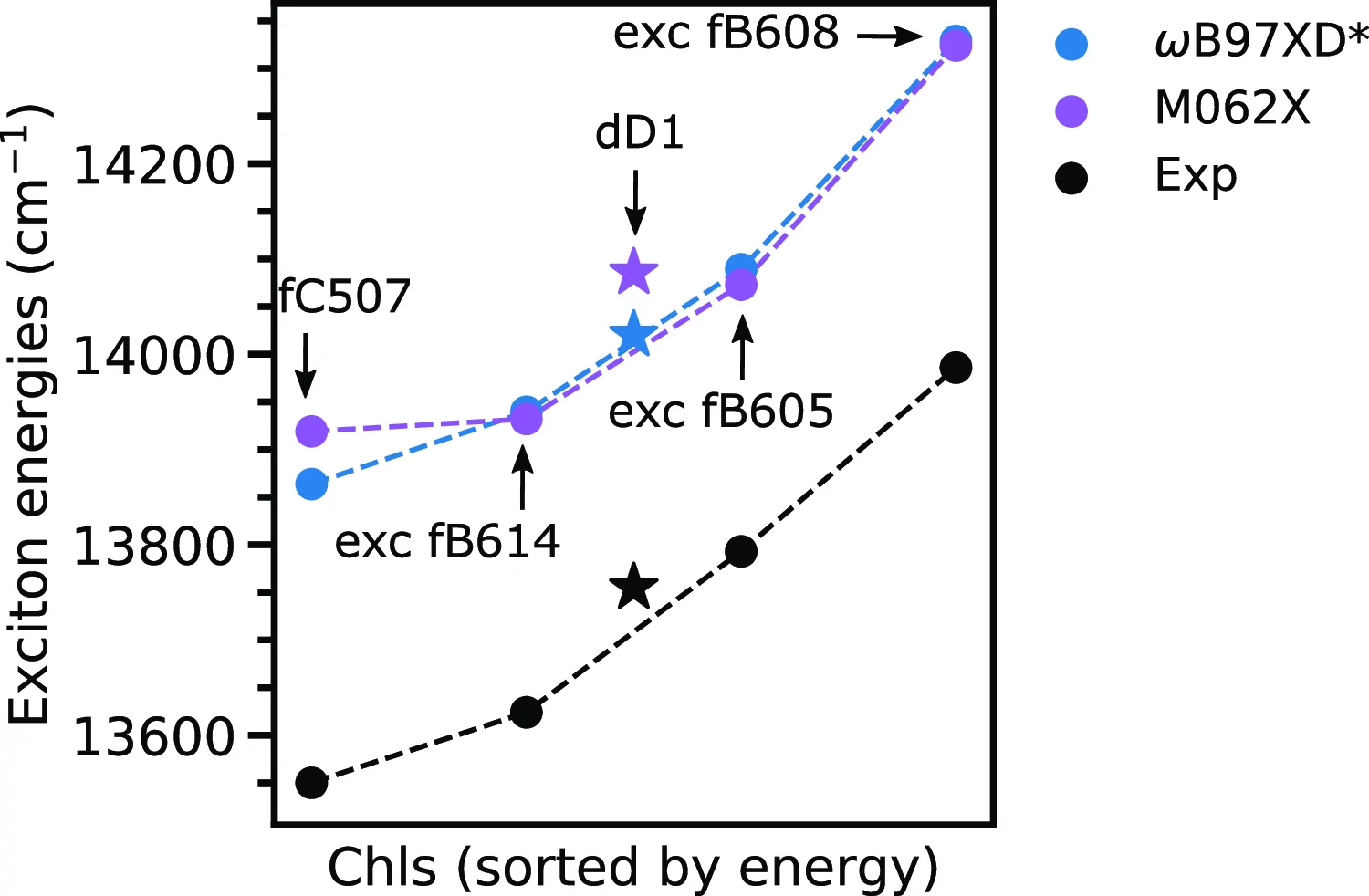
Comparison of the calculated exciton energies with the energies extracted from 2DES measurements of the far-red PSII complex at 77 K (ref 10). The calculated energies were shifted by constant offsets of 1800 and 930 cm^–1^ for the M06–2X and ωB97X-D* functionals, respectively, to facilitate comparison of the relative energy spacings.

The spectroscopic signature of Chl *d*D1 can be understood also from the contributions in Figure 6, which account for the actual line shape. As the Chl *d*D1 transition is essentially uncoupled, its contribution to the absorption spectrum can be directly compared to the bleaching at 727 nm obtained by inducing charge separation in the RC.^10^ In the M06–2X spectrum, the Chl *d*D1 absorption maximum appears at ∼13,900 cm^–1^ (720 nm), slightly too blue-shifted with respect to the experiments. On the other hand, for ωB97X-D* the Chl *d*D1 maximum is around 13800 cm^–1^ (725 nm), very close to the measured one (see Table S8).

### 3.6 Reliability of the Chl *f* Assignment

The very recent structure of *C. thermalis* FR-PSII obtained by Leong et al.^26^ suggested a different assignment for Chl *f* positions in CP47. Based on ESP cone scans and on the lack of H-bonding partners of Chl B605, the authors of ref 26. assigned this position to a Chl *a*, and instead suggested position B617 as a Chl *f*. This assignment was supported by mutagenesis experiments for *C. thermalis* FR-PSII, raising the question of the Chl *f* assignment used in ref 23. In the following, we discuss the reliability of this assignment.

On the one hand, our results convincingly reproduce the main features of the far-red absorption band, as well as the ladder of excitonic energies extracted from 2DES experiments.^10^ As such, the current assignment with B605 being a Chl *f* cannot be ruled out on the basis of our calculations. Additionally, the lowest-energy peak of *C. thermalis* FR-PSII at 750 nm, assigned in ref 26. to Chl *f*B617 is not present in *Synechococcus* FR-PSII.^10^ Therefore, the assignment by Gisriel et al.^23^ may still be correct for *Synechococcus*, if not for *C. thermalis*. This is supported by the fact that Glu107, the H-bonding residue to Chl B617, is not conserved in *Synechococcus*.^26^ On the other hand, if Chl B605 were indeed a Chl *a* in *Synechococcus* FR-PSII, another Chl *f* would be needed to explain the absorption at around 14,000 cm^–1^. It is nonetheless possible for the Chl B605 to be a Chl *a*, provided that another Chl *f* with a similar environment exists in *Synechococcus* FR-PSII.

A possible argument *against* the assignment of Chl B605 as a Chl *f* comes from the unusual dipole arrangement in the B605–B608–B614 triad. As explained above, this dipole arrangement gives rise to a redistribution of the dipole strength toward higher excitons, which renders the lowest exciton of CP47 rather optically dark. This feature appears inconsistent with the necessity of efficient energy transfer toward Chl *d*D1 in the RC.

A definite assignment of the Chl *f* locations in CP47 is therefore still missing, at least for *Synechococcus* FR-PSII. Measurements of the superradiance of the lowest CP47 state or mutagenesis experiments could confirm or rule out the presence of Chl *f* at the B605 position. Having said all this, our main conclusions about the lowest-energy sites in both CP47 and CP43 are unaffected by the assignment of B605.

## 4 Conclusions

We have presented a first-principles characterization of the excitonic landscape of the far-red PSII from *Synechococcus* sp. PCC 7335 by combining QM/MM calculations of chlorophyll site energies, excitonic couplings, and a detailed description of environmental effects, including polarization. Benchmark calculations against SOS-CC2 and SCS-CC2 reference data demonstrate that density functionals with sufficient exchange contribution (M06–2X and ωB97X-D*) provide a reliable description of the relative energetics of Chl *a*, Chl *d*, and Chl *f*. However, different functionals give different systematic errors for different types of Chls, which impacts the assignment of low-energy sites. Our results show that care must be taken in selecting a reliable excited-state method for far-red chlorophylls, and benchmarks based purely on gas-phase calculations are not sufficient.

Overall, our calculations enable the construction of a physically sound excitonic Hamiltonian that describes the electronic structure and optical properties of FR-PSII. We clearly show that the protein electrostatic environment is the dominant factor governing the energetic tuning of the far-red chlorophylls, whereas structural distortions play a comparatively minor role. Electrostatic determinants are not limited to one or a few amino acids, but involve multiple residues around each Chl. Environment polarization gives rise to a largely uniform red shift of the pigment excitation energies while having only a limited effect on their relative ordering. Excitonic interactions are found to be significant only among the subset of the CP47 Chl *f* pigments, whereas the reaction-center Chl *d* remains largely localized.

Remarkably, the simulated absorption spectra reproduce the experimental line shape, and the calculated exciton energies closely follow the sequence of states identified by 2DES.^10^ This agreement supports the assignment of the experimentally observed low-energy excitons to specific Chl *f* and Chl *d* pigments.

Beyond providing a molecular interpretation of the far-red absorption band, the present work establishes a quantitative framework for future simulations of excitation-energy transfer and primary charge separation in far-red photosystems. Such studies are essential for understanding how oxygenic photosynthesis remains efficient despite the reduced energetic budget imposed by far-red photons.

Finally, we note that additional cryo-EM structures of FR-PSII have recently become available^26^ and suggest alternative assignments for some Chl *f* binding sites. Whether these alternative assignments are valid also for the present *Synechococcus* FR-PSII remains an open question, given the spectroscopic difference between the two species. Applying the methodology developed here to these new structural models will provide an important test for the assignment of the energetic landscape in different far-red PSII. Further efforts in this direction are ongoing in our group.

## Supplementary Material



## Data Availability

All data needed to evaluate the conclusions in this paper are present in the paper and/or the Supporting Information.

## Notes

Difference ESP of isolated chlorophylls; electronic coupling data; details on spectral densities; additional data on isolated chlorophylls; additional data for chlorophylls in the protein; character of the excitonic states; absorption spectra for the uncoupled case; assignment of exciton states; additional tests on absorption spectra; and energy transfer rate calculations (PDF)
