# Supplementary material for: Assigning the Low-Energy Excitons in Far-Red Photosystem II through Atomistic Modeling
Source: J Phys Chem B. 2026 Oct 21;130(44):11746–56. doi: 10.1021/acs.jpcb.6c05218 (PMC13746781; doi:10.1021/acs.jpcb.6c05218)
Supplement: Supplementary file 1 [file jp6c05218_si_001.pdf]

# Supporting Information: Assigning the Low-Energy Excitons in Far-Red Photosystem II through Atomistic Modeling

Samuele Giannini*[1], Luca Ancillotti[1], and Lorenzo Cupellini*[1]

[1]Dipartimento di Chimica e Chimica Industriale, Università di Pisa, via G. Moruzzi 13, 56124 Pisa, Italy

*Corresponding Author, e-mail: samuele.giannini@unipi.it; lorenzo.cupellini@unipi.it

# S1 Chemical structure and difference ESP of isolated chlorophylls

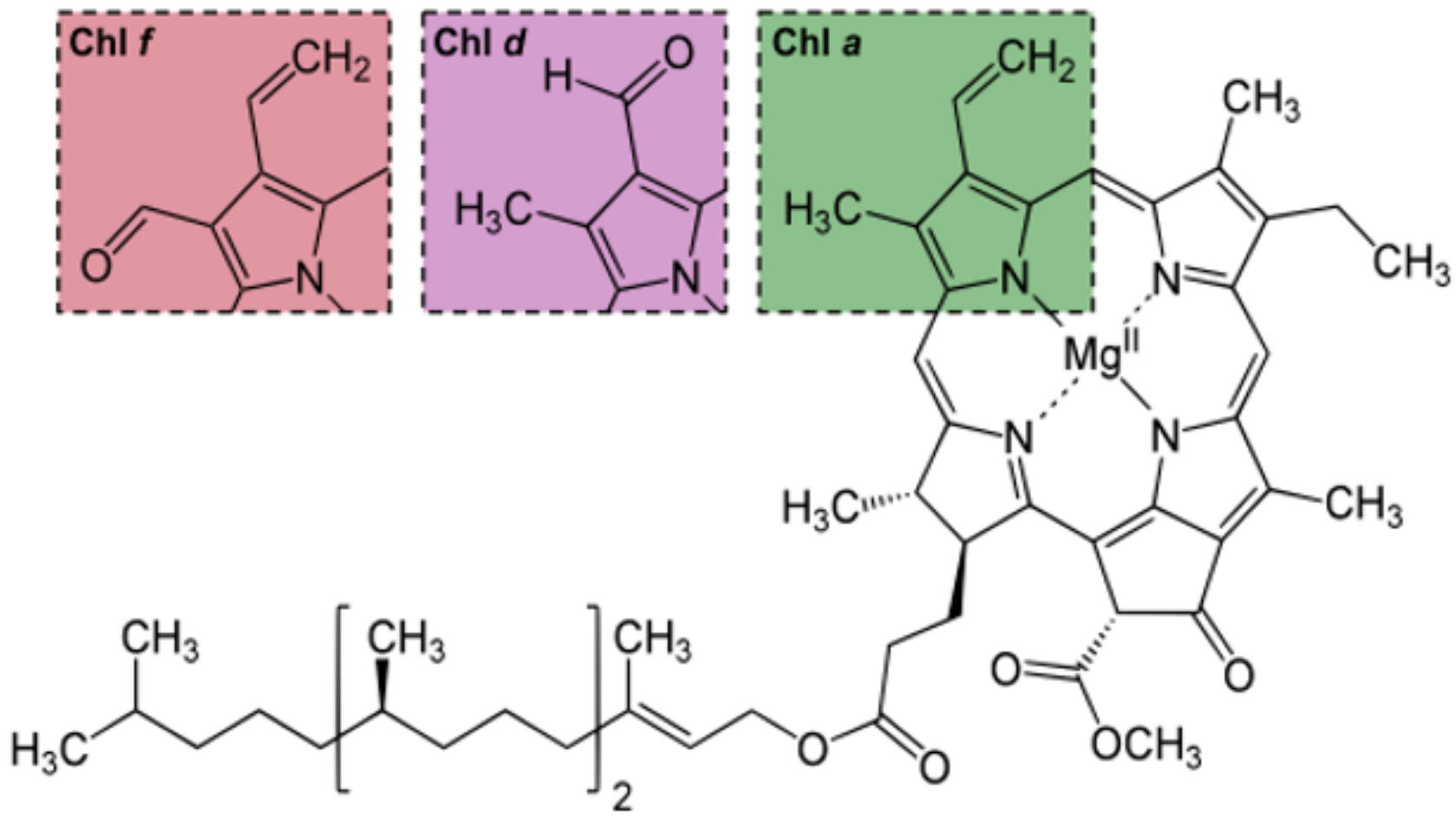


**Figure S1:** Chemical structures of Chl $d$, $f$ investigated in this work, compared to the canonical Chl $a$.

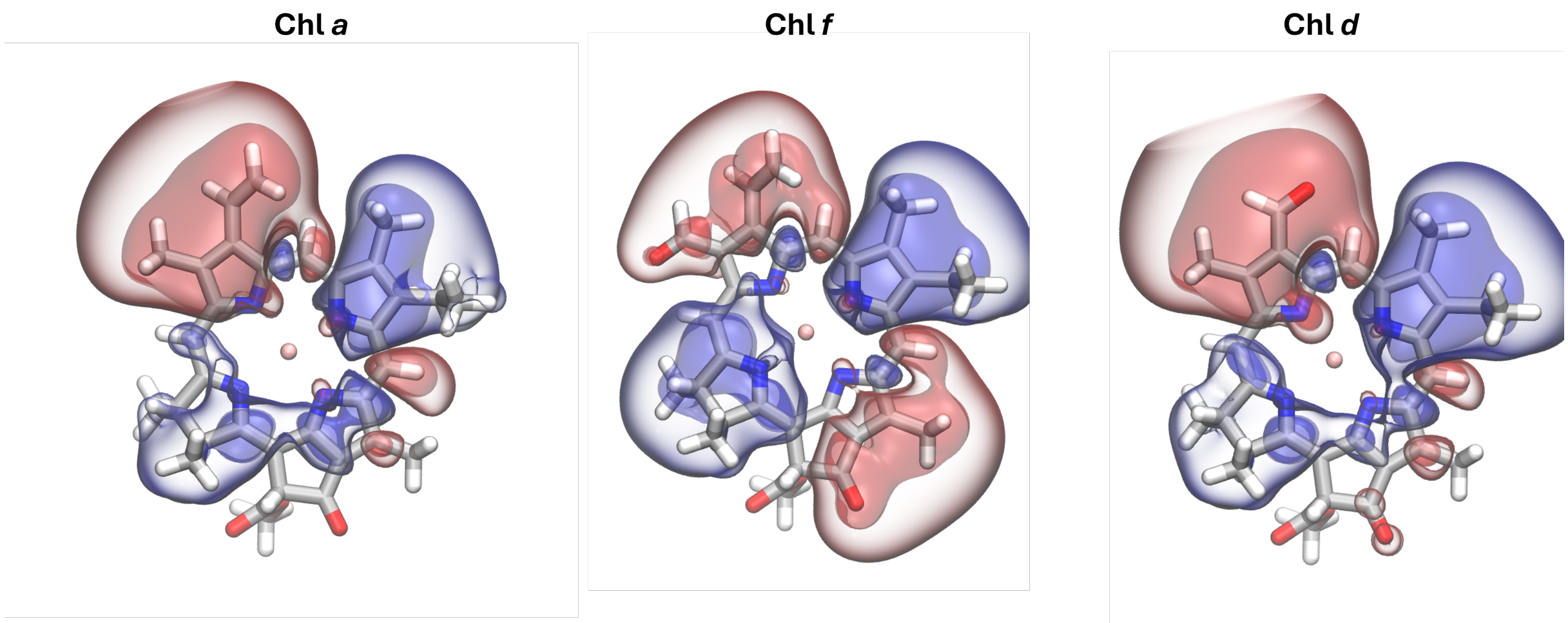


**Figure S2:** Difference electrostatic potential (excited-minus-ground state) for Chls *a,d,f* calculated *in vacuo* with the $\omega$B97X-D*/6-31G(d) level.

# S2 Electronic couplings

A comparison between the coupling obtained with different functionals and basis-set is reported in Table S1 and Table 2 of the main text.

**Table S1:** Excitonic couplings ($cm^{-1}$) using the $\omega$B97X-D* functional and different basis sets.

| Chl 1 | Chl 2 | 6-31G(d) | def2-TZVP |
|---|---|---|---|
| dD1 | fB605 | 0.2 | 0.1 |
| dD1 | fB608 | -2.0 | -2.2 |
| dD1 | fB614 | -2.7 | -3.0 |
| dD1 | fC507 | 5.1 | 5.5 |
| fB605 | fB608 | 115.5 | 121.3 |
| fB605 | fB614 | -50.1 | -46.5 |
| fB605 | fC507 | 0.3 | 0.4 |
| fB608 | fB614 | 58.6 | 64.2 |
| fB608 | fC507 | -0.3 | -0.3 |
| fB614 | fC507 | -0.3 | -0.4 |

# S3 Spectral densities

The interaction between electronic excitation and nuclear motion was described using the spectral density formalism, which provides a compact representation of vibronic coupling. Within the harmonic approximation, the ground- and excited-state potential energy surfaces are assumed to possess identical curvatures while differing in their equilibrium geometries. Under these assumptions, the spectral density is constructed from the normal vibrational modes of the chromophore, with each mode contributing according to its frequency and associated reorganization energy. The total reorganization energy is obtained as the sum of the individual modal contributions and corresponds to the relaxation energy accompanying nuclear reorganization after vertical excitation as explained in previous works.[S1]

Here, we obtained the spectral density at the QM/MM level to get the normal-mode frequencies of the ground state at B3LYP/6-31G(d) level of theory, while excited-state gradients at the Franck–Condon geometry were used to estimate the mode-specific reorganization energies using CAM-B3LYP/6-31G(d) with $\omega = 0.2$. Moreover, the spectral density was modeled by a sum of an overdamped Brownian oscillator (BO)[S2] plus the underdamped modes obtained from the normal-mode analysis. As BO model parameters[S1] we used $\gamma = 40$, $\lambda_{i,env} = 20$ cm$^{-1}$. The sensitivity to these parameters was tested by repeating the spectral calculations with $\lambda_{\text{env}} = 40\,\text{cm}^{-1}$ and with $\gamma = 80\,\text{cm}^{-1}$, while keeping the other parameter fixed at its reference value Fig. S14.

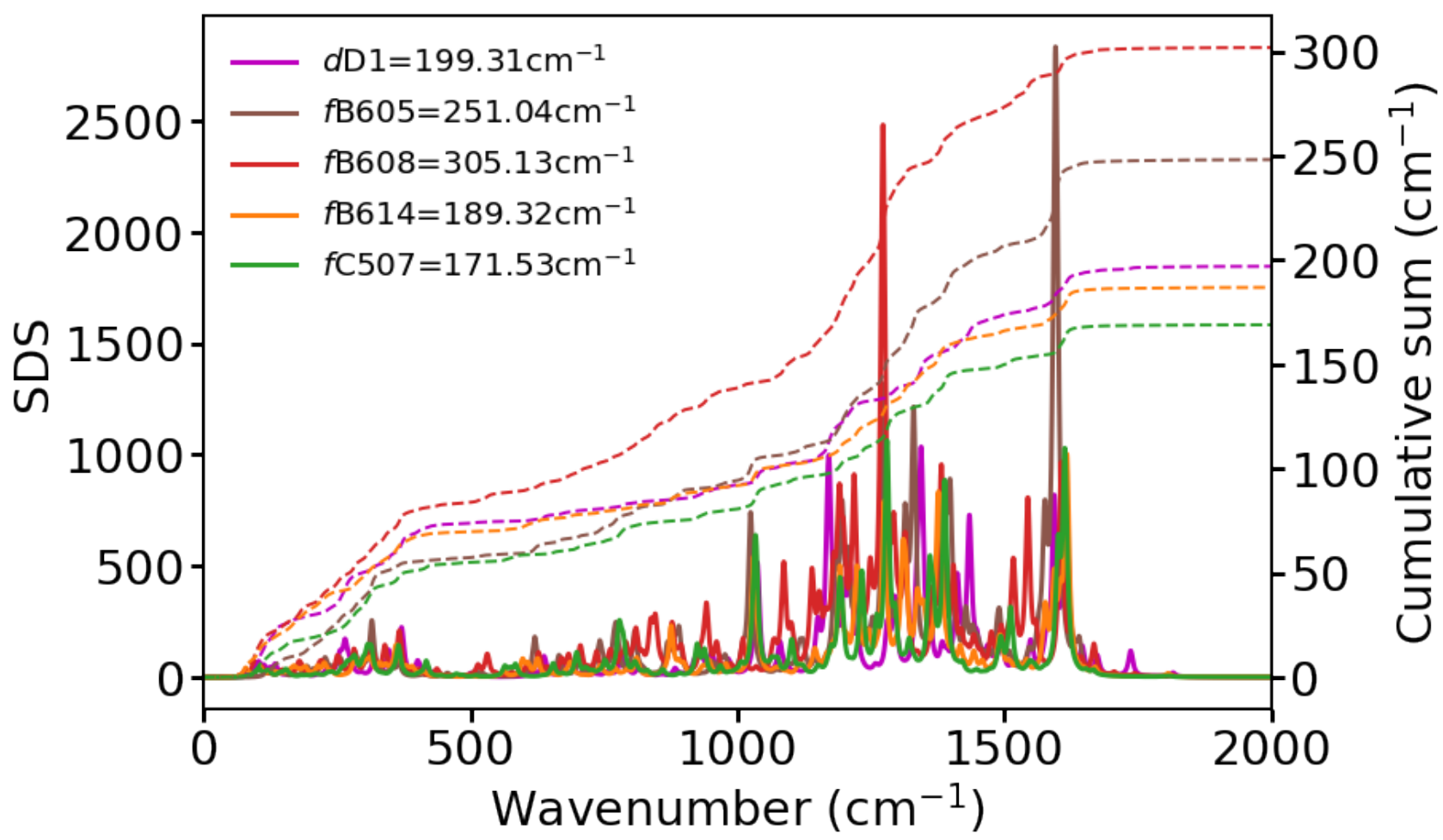


**Figure S3:** Spectral Densities for the different Chls and related cumulative sum on the second axis.

# S4 Additional Data for Isolated Chlorophylls

**Table S2:** Vertical excitation energies ($cm^{-1}$) calculated using different density-functional and post-HF methods in vacuo and in methanol represented by the PCM model. All data refer to the 6-31G(d) basis set, except SOS-CC2 and SCS-CC2 which refer to def2TZVP basis. The reference geometries for the different Chls are obtained at the B3LYP/6-31G(d) level in PCM methanol.

| $\tilde{\nu}$ ($cm^{-1}$) | B3LYP | CAM-B3LYP | CAM-B3LYP $\omega = 0.2$ | LC-BLYP | LC-BLYP $\omega = 0.195$ | M06-2X | $\omega$B97X-D | $\omega$B97X-D* $\omega = 0.3$ | SCS-CC2 | SOS-CC2 |
|---|---|---|---|---|---|---|---|---|---|---|
| **In vacuo** | | | | | | | | | | |
| Chl $a$ | 17328 | 17017 | 17179 | 15844 | 16826 | 17462 | 16769 | 16612 | 16895 | 16618 |
| Chl $d$ | 16677 | 16212 | 16458 | 14582 | 16054 | 16721 | 15904 | 15695 | 16297 | 15901 |
| Chl $f$ | 16544 | 16217 | 16396 | 14783 | 16021 | 16676 | 15925 | 15746 | 16049 | 15640 |
| **Methanol (PCM)** | | | | | | | | | | |
| Chl $a$ | 16990 | 16746 | 16876 | 15737 | 16555 | 17175 | 16521 | 16385 | – | – |
| Chl $d$ | 16167 | 15783 | 15997 | 14354 | 15631 | 16293 | 15498 | 15337 | – | – |
| Chl $f$ | 16022 | 15740 | 15904 | 14411 | 15562 | 16189 | 15471 | 15313 | – | – |

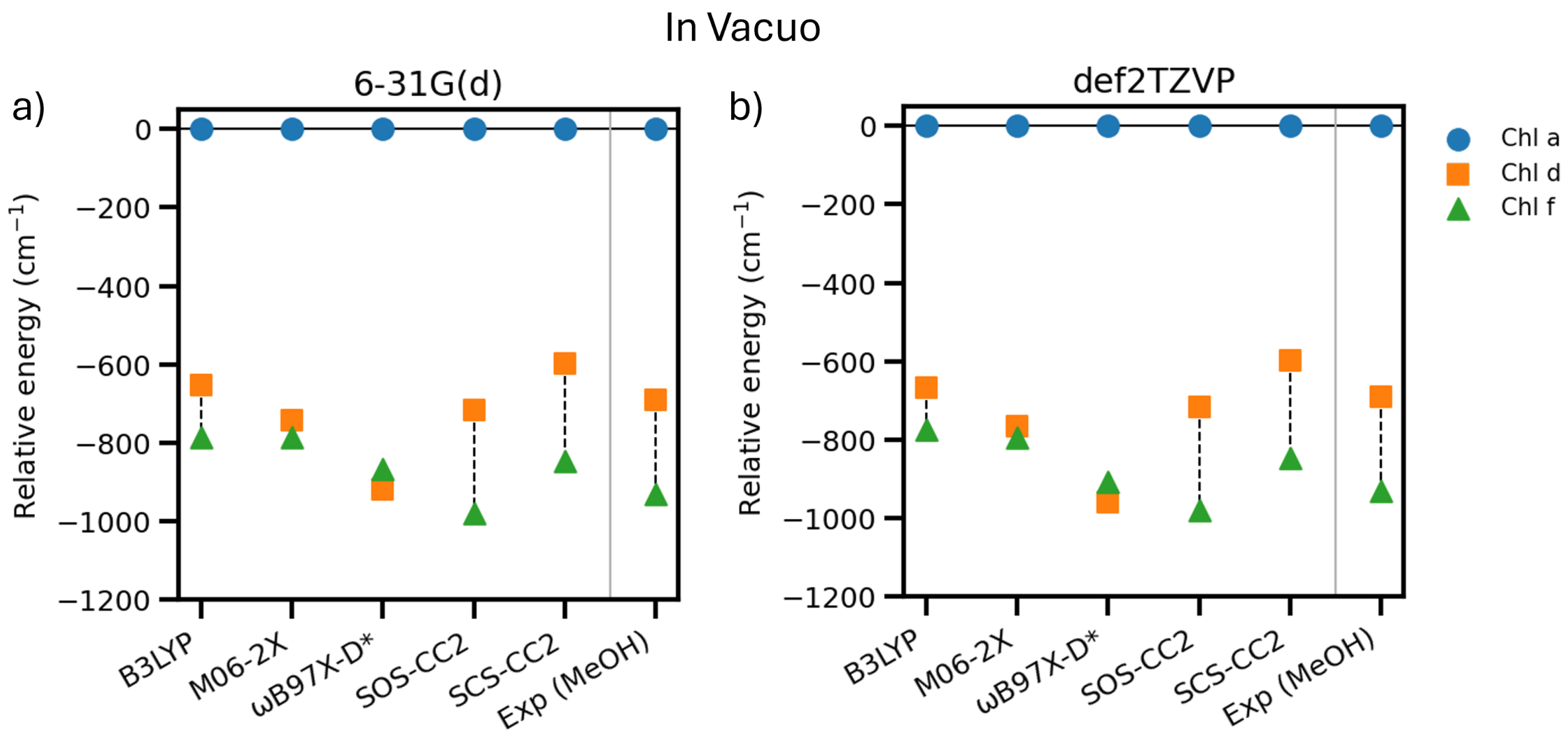


**Figure S4:** $Q_y$ excitation energies differences for Chl $a$, $f$, $d$ calculated in the gas phase with 6-31G(d) and def2TZVP basis-set at geometries optimized at the B3LYP/6-31G(d) level in PCM methanol. SCS-CC2 and SOS-CC2 results refer to the def2TZVP basis-set.

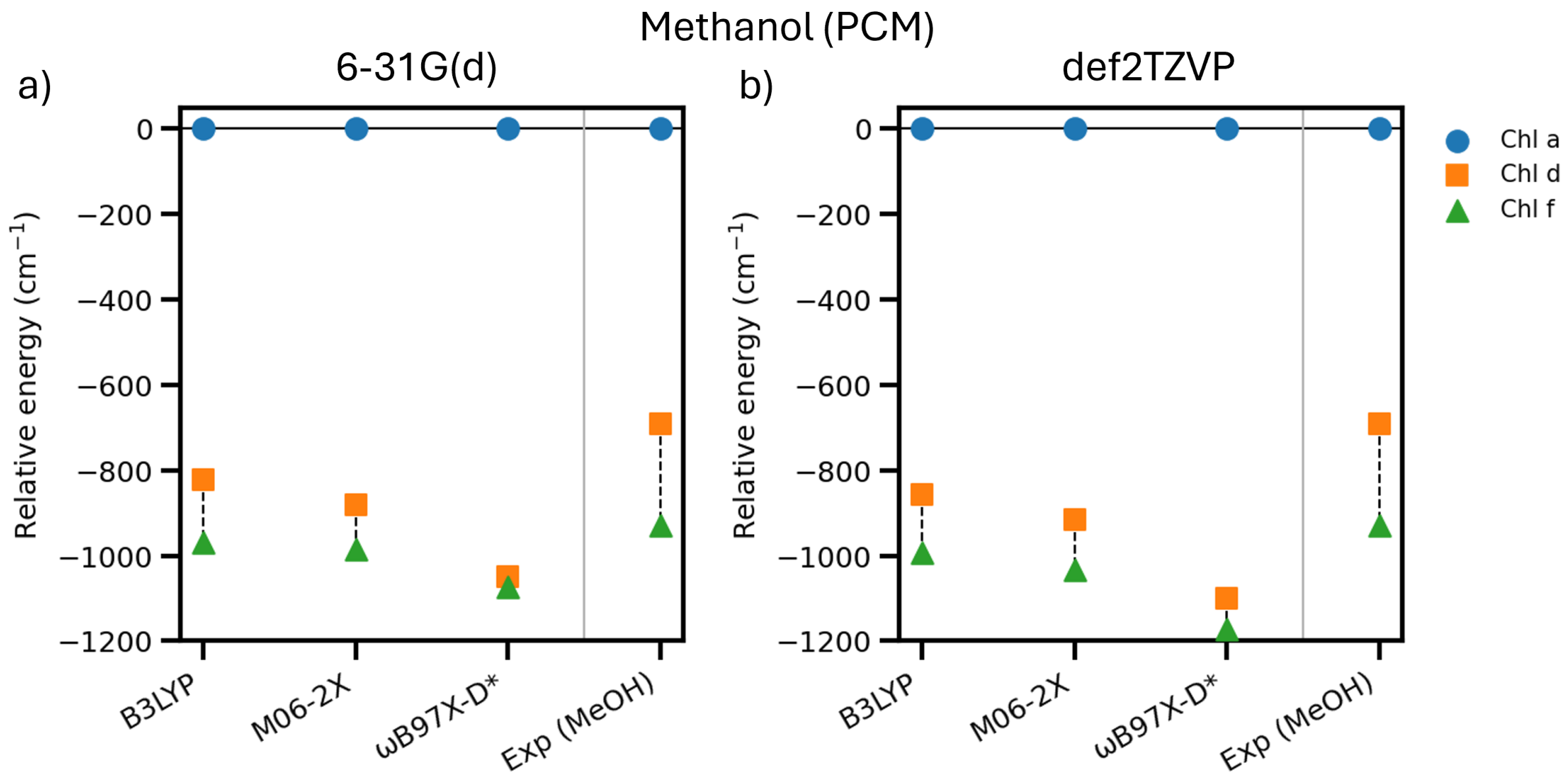


**Figure S5:** $Q_y$ excitation energies differences for Chl $a$, $f$, $d$ calculated in methanol with PCM with 6-31G(d) and def2TZVP basis-set at geometries optimized at the B3LYP/6-31G(d) level in PCM methanol.

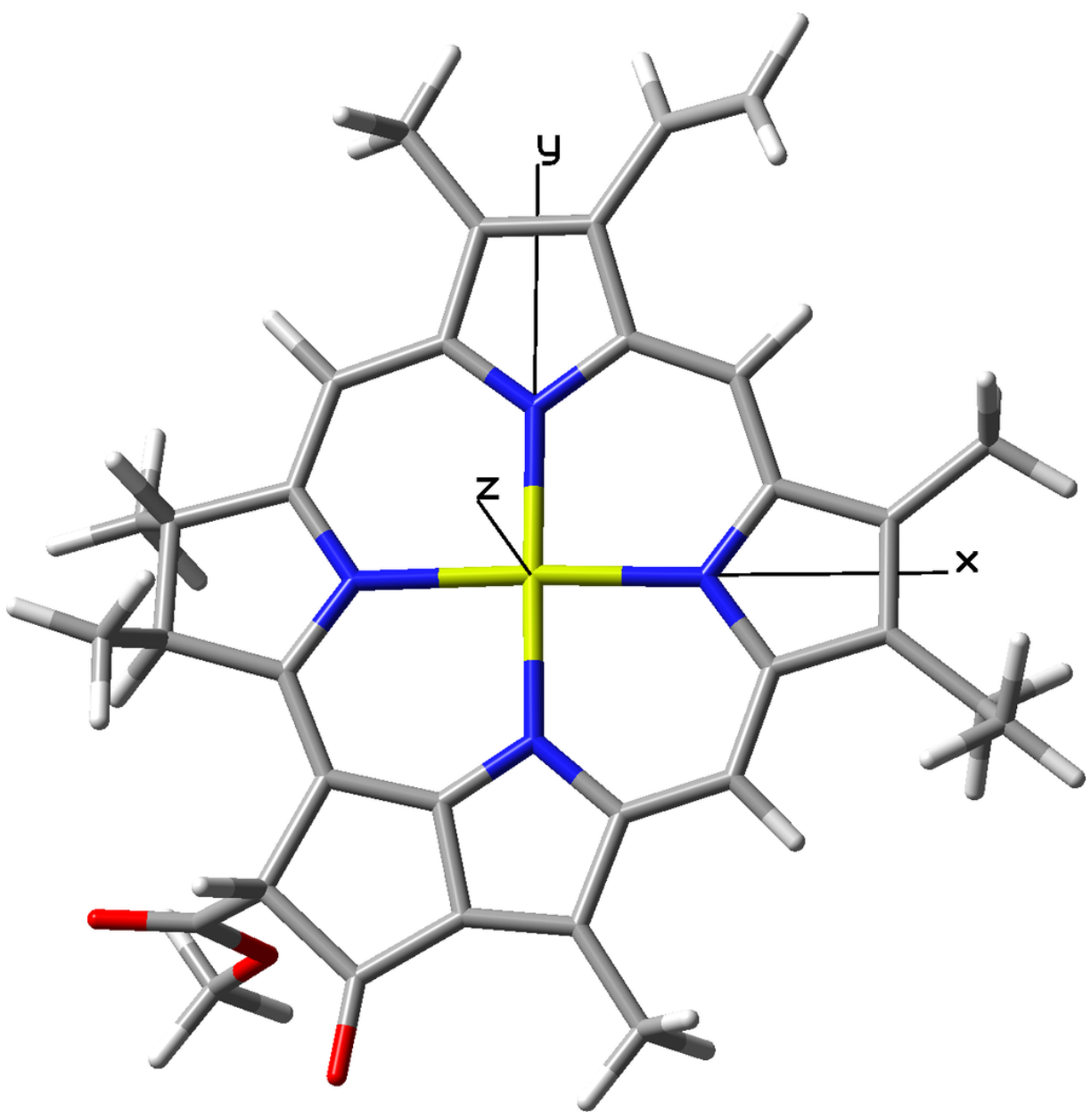


**Figure S6:** Alignment of Chl $a$ on the cartesian axes employed for the results in Table S3.

**Table S3:** Electric field effect on Chl excitation energies calculated at the $\omega$B97X-D*/6-31G(d) level. Change in excitation energy ($cm^{-1}$) of each Chl type upon applying a homogeneous electric field of 0.01 $e$ bohr$^{-2}$ (0.51 V/Å) in the given direction. The Chl molecule is in the $xy$ plane, with the $N_C$ and $N_D$ atoms in the positive $x$ and $y$ directions (Figure SXX). The geometry of each Chl was optimized in gas phase at the B3LYP/6-31G(d) level.

| Field direction | Chl *a* | Chl *d* | Chl *f* |
|---|---|---|---|
| $+x$ | 56 | 162 | 15 |
| $-x$ | −92 | −144 | −100 |
| $+y$ | −543 | −77 | 319 |
| $-y$ | 411 | 727 | 295 |
| $+z$ | −29 | 31 | −32 |
| $-z$ | 23 | −33 | 25 |

# S5 Errors in Relative Chl $f$–Chl $d$ Excitation Energies

The QM/MM mean signed errors, 185 $cm^{-1}$ for M06-2X and 330 $cm^{-1}$ for $\omega$B97X-D*, are close to the corresponding isolated-pigment errors of 203 and 299 $cm^{-1}$, respectively. This agreement supports the approximate transferability of the isolated-pigment correction to FR-PSII, within an uncertainty of about 30 $cm^{-1}$.

**Table S4:** Signed errors in the Chl $f$–Chl $d$ excitation-energy gaps predicted using the M06-2X and $\omega$B97X-D* functionals, relative to the corresponding SCS-CC2 reference values. Results are reported for the isolated pigments (*in vacuo*) and for each Chl $f$ pigment relative to Chl $d_{\mathrm{D1}}$ in the FR-PSII protein environment (QM/MM). The mean signed error (MSE) is calculated over the four QM/MM pigment pairs. All values are given in $cm^{-1}$.

| Energy gap | M06-2X | $\omega$B97X-D* |
|---|---|---|
| *In vacuo* | | |
| Chl $f$–Chl $d$ | 203 | 299 |
| QM/MM | | |
| Chl $f_{\mathrm{B605}}$–Chl $d_{\mathrm{D1}}$ | 117 | 286 |
| Chl $f_{\mathrm{B608}}$–Chl $d_{\mathrm{D1}}$ | 93 | 273 |
| Chl $f_{\mathrm{B614}}$–Chl $d_{\mathrm{D1}}$ | 296 | 421 |
| Chl $f_{\mathrm{C507}}$–Chl $d_{\mathrm{D1}}$ | 234 | 340 |
| Mean signed error | 185 | 330 |

# S6 Additional Data for Chlorophylls in the Protein

**Table S5:** Vertical excitation energies ($cm^{-1}$) of the five far-red chlorophylls computed using different density functionals and the 6-31G(d) basis set. Results are reported for the isolated pigments (in vacuo), electrostatic QM/MM, and polarizable QM/MM (QM/MMPol), all evaluated at the QM/MM-optimized geometries.

| $\tilde{\nu}$ ($cm^{-1}$) | B3LYP | CAM-B3LYP | CAM-B3LYP $\omega = 0.2$ | LC-BLYP | LC-BLYP $\omega = 0.195$ | M06-2X | $\omega$B97X-D | $\omega$B97X-D* $\omega = 0.3$ |
|---|---|---|---|---|---|---|---|---|
| **In vacuo** | | | | | | | | |
| dD1 | 16690 | 16200 | 16461 | 14466 | 16053 | 16711 | 15882 | 15663 |
| fB605 | 16407 | 16136 | 16288 | 14809 | 15923 | 16572 | 15858 | 15704 |
| fB608 | 16524 | 16208 | 16380 | 14797 | 16004 | 16667 | 15915 | 15730 |
| fB614 | 16534 | 16201 | 16388 | 14700 | 16009 | 16665 | 15898 | 15716 |
| fC507 | 16421 | 16107 | 16279 | 14710 | 15910 | 16554 | 15823 | 15626 |
| **QM/MM** | | | | | | | | |
| dD1 | 16300 | 15840 | 16099 | 14045 | 15686 | 16343 | 15523 | 15278 |
| fB605 | 16175 | 15988 | 16108 | 14696 | 15736 | 16448 | 15700 | 15552 |
| fB608 | 16193 | 16076 | 16146 | 14892 | 15808 | 16543 | 15792 | 15658 |
| fB614 | 16214 | 15875 | 16069 | 14332 | 15678 | 16295 | 15575 | 15355 |
| fC507 | 16105 | 15823 | 15986 | 14379 | 15607 | 16275 | 15525 | 15316 |
| **QM/MMPol** | | | | | | | | |
| dD1 | 15632 | 15175 | 15446 | 13348 | 15060 | 15683 | 14863 | 14649 |
| fB605 | 15590 | 15418 | 15533 | 14205 | 15198 | 15875 | 15145 | 15043 |
| fB608 | 15695 | 15586 | 15684 | 14407 | 15365 | 16058 | 15313 | 15202 |
| fB614 | 15687 | 15384 | 15570 | 13907 | 15215 | 15800 | 15097 | 14921 |
| fC507 | 15581 | 15272 | 15451 | 13814 | 15097 | 15731 | 14983 | 14793 |

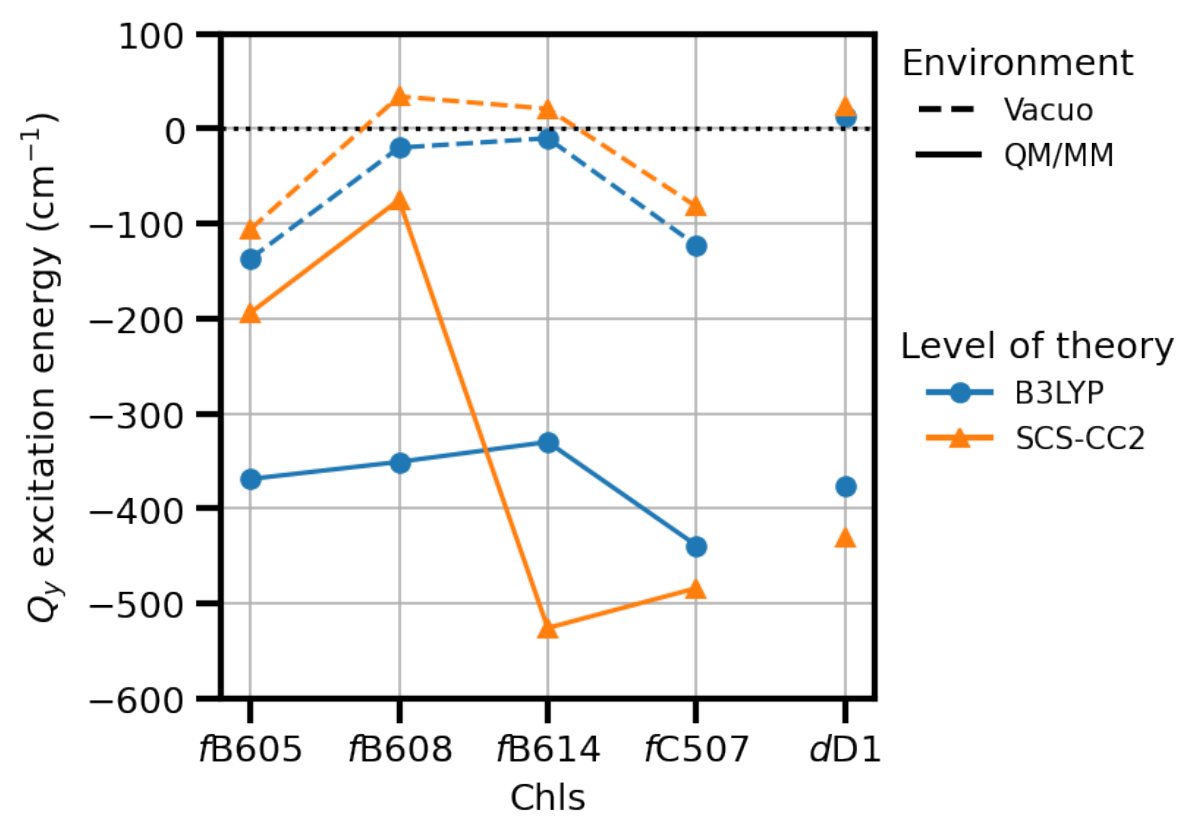


**Figure S7:** Comparison of the changes in the $Qy$ excitation energies of the far-red chlorophylls induced by geometry (dashed lines) and by the protein environment (solid lines) calculated with B3LYP and SCS-CC2. Energies are given relative to the isolated pigments optimized in vacuo (horizontal dotted line). While both methods predict small effects from geometrical distortions, B3LYP fails to reproduce the site-specific electrostatic tuning predicted by the SCS-CC2 reference calculations.

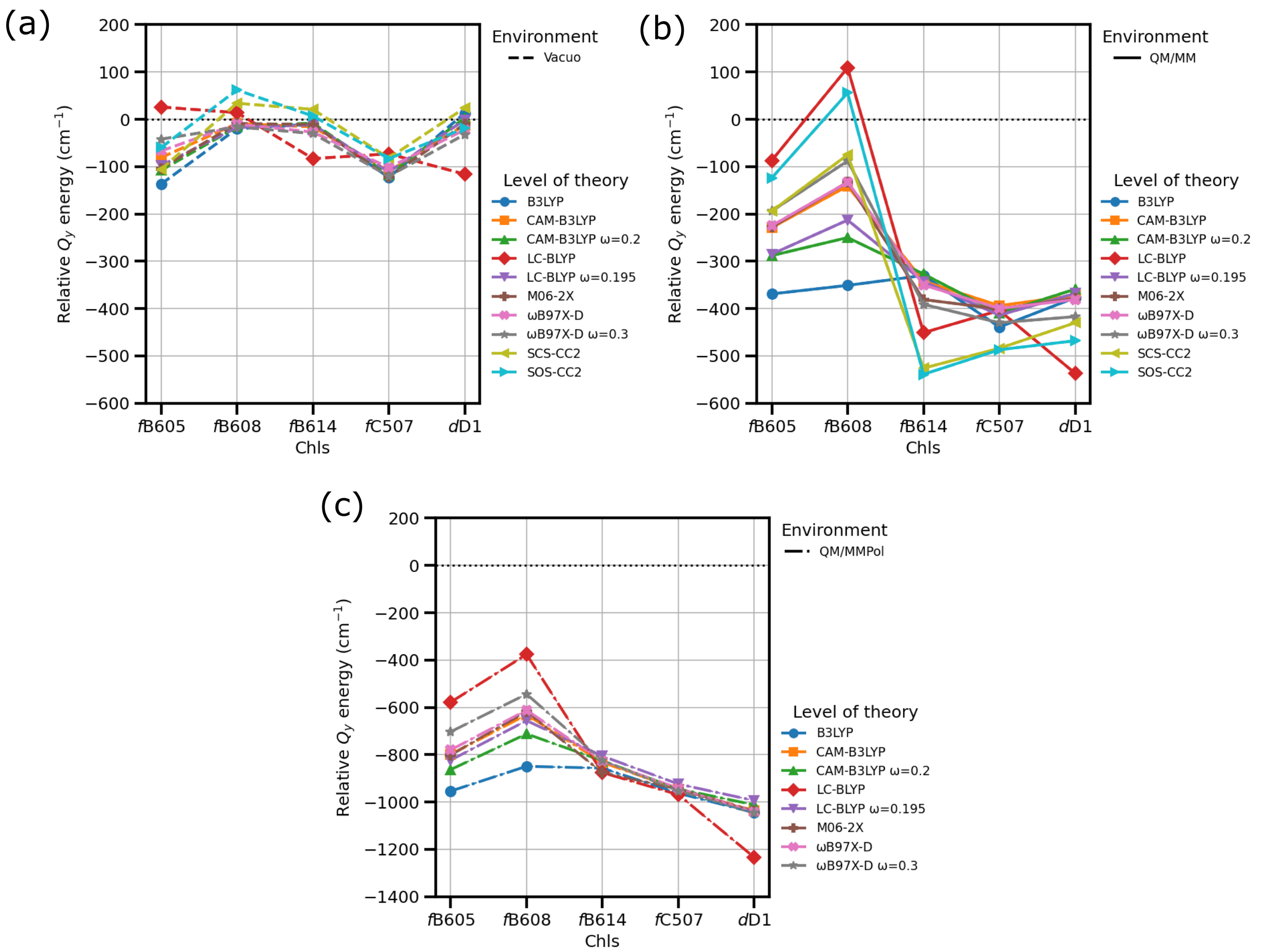


**Figure S8:** Changes in the Q$y$ excitation energies of the four Chl f pigments (fB605, fB608, fB614, and fC507) and the reaction-center Chl d (dD1) calculated with different exchange-correlation functionals, with respect to the same Chls in gas phase. Panel (a) reports excitation energies of the isolated pigments extracted from the QM/MM-optimized geometries (in vacuo), thereby accounting only for geometrical distortions. Panels (b) and (c) show the corresponding QM/MM and QM/MMPol excitation energies, including the electrostatic and electrostatic plus polarization effects of the protein environment, respectively. Energies are given relative to the corresponding excitation energies of the isolated pigments optimized *in vacuo* (horizontal dotted line), analogously to Figure 2a of the main text.

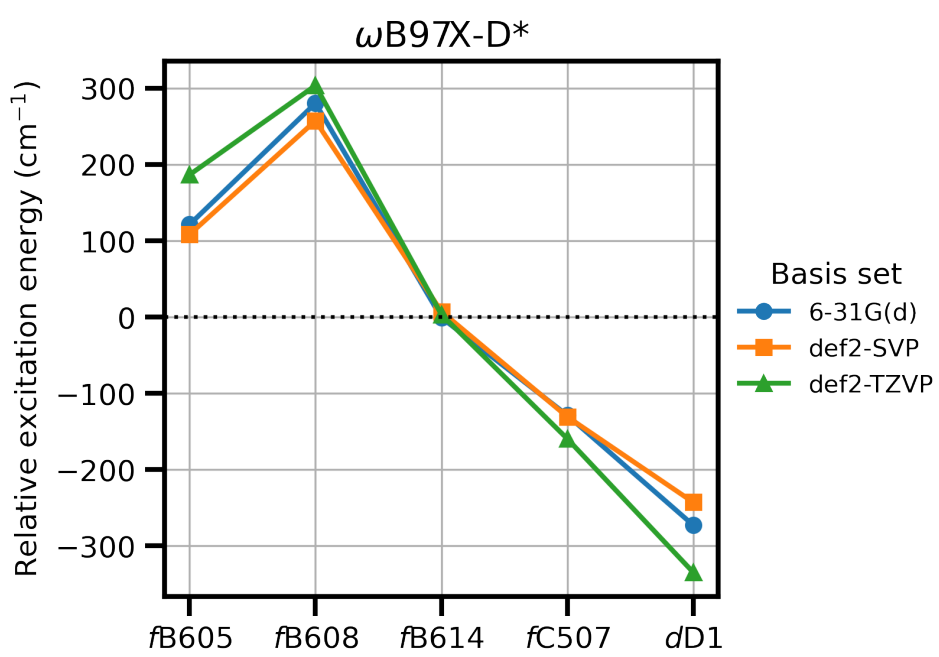


**Figure S9:** Relative excitation energies at QM/MMpol level of theory of the chlorophylls computed with the ωB97X-D* functional using the 6-31G(d), def2-SVP, and def2-TZVP basis sets. For each basis set, the energies have been shifted by subtracting the corresponding average excitation energy, highlighting the relative energetic ordering of the pigments.

**Table S6:** Comparison between the electrochromic shift calculated at the QM/MM level (QM/MM *minus* gas-phase, at the in-protein geometry) and the CDC prediction of the electrochromic shift. These data refer to the $\omega$B97X-D*/6-31G(d) level of theory.

| Chl | QM/MM | CDC |
|---|---|---|
| dD1 | −385 | −341 |
| fB605 | −152 | −36 |
| fB608 | −72 | −90 |
| fB614 | −361 | −437 |
| fC507 | −310 | −298 |

# S7 Additional Data on Electrostatic contributions

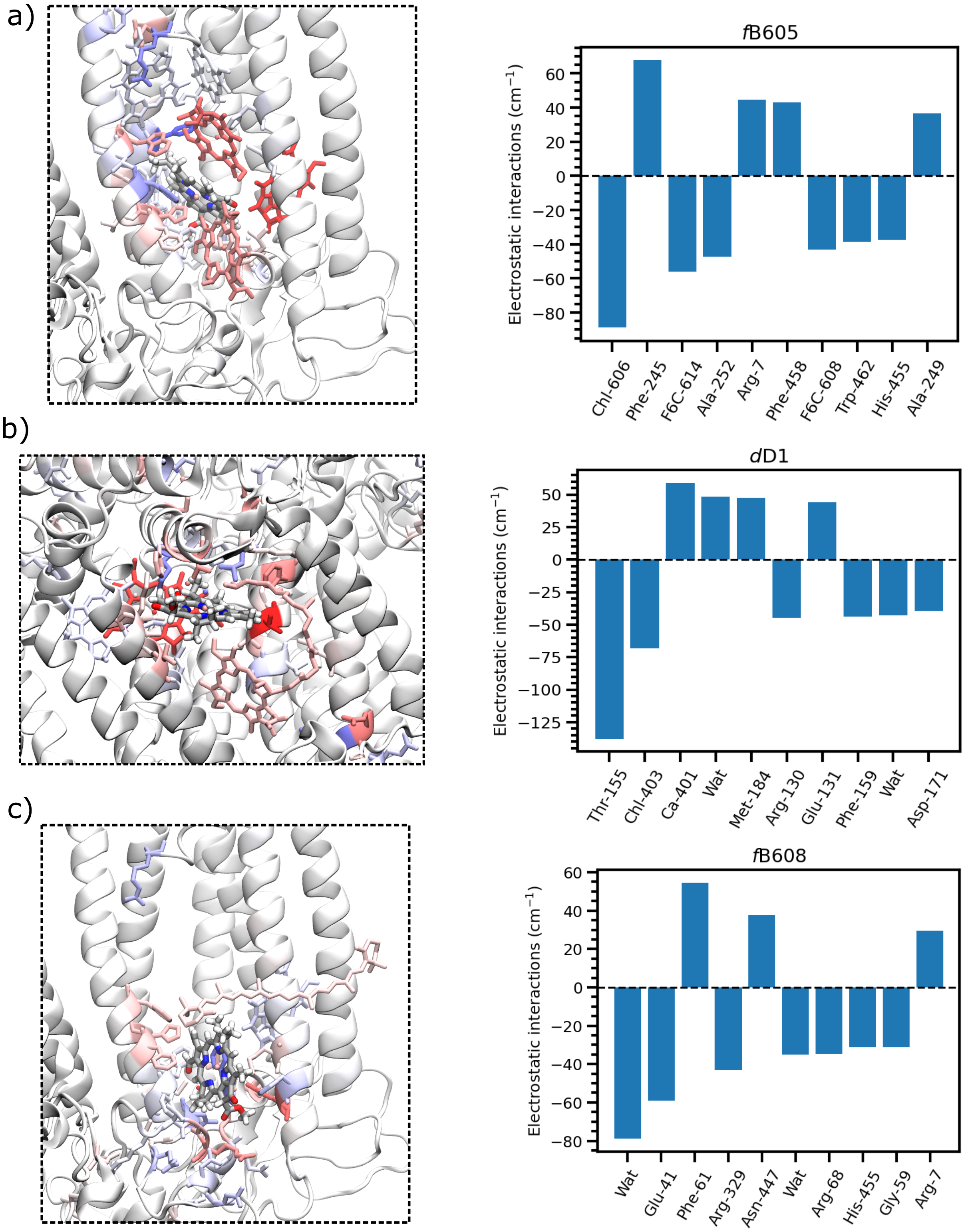


**Figure S10:** Contributions of residues and cofactors to the electrochromic shifts. (a) Chl *f*B605 (b) Chl *d*D1 (e) Chl *f*B608. Red/blue colors indicate residues with a negative/positive contribution. Phytol tails of the Chls have been omitted for clarity.

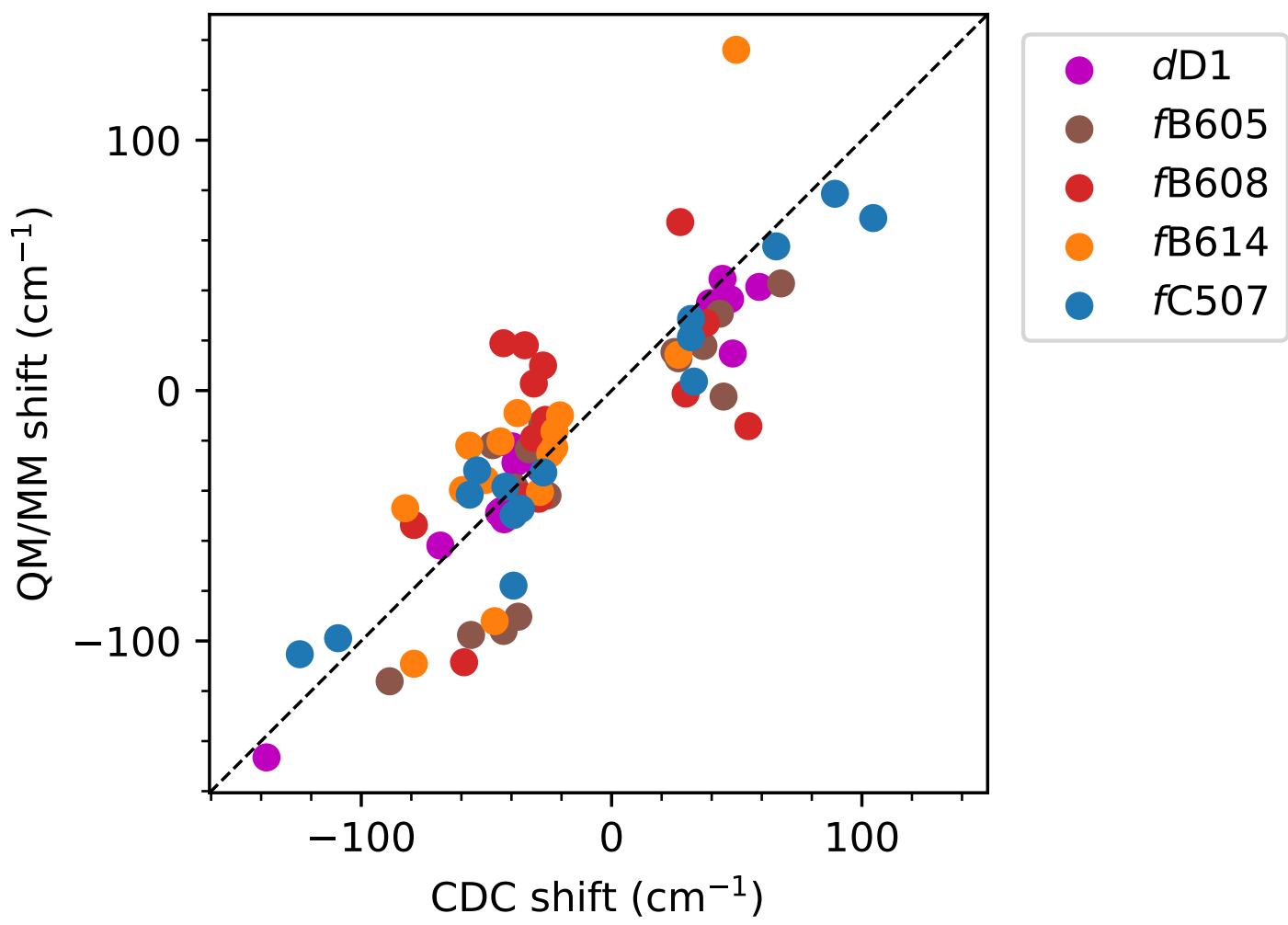


**Figure S11:** Comparison of the residue contributions obtained with the CDC method with the ones calculated at the QM/MM level. The latter were obtained by turning off each selected residue and calculating the QM/MM excitation energy with *vs* without the residue.[S3] Calculations were performed for the 15 residues with largest contributions according to the CDC estimation.

# S8 Excitonic interactions

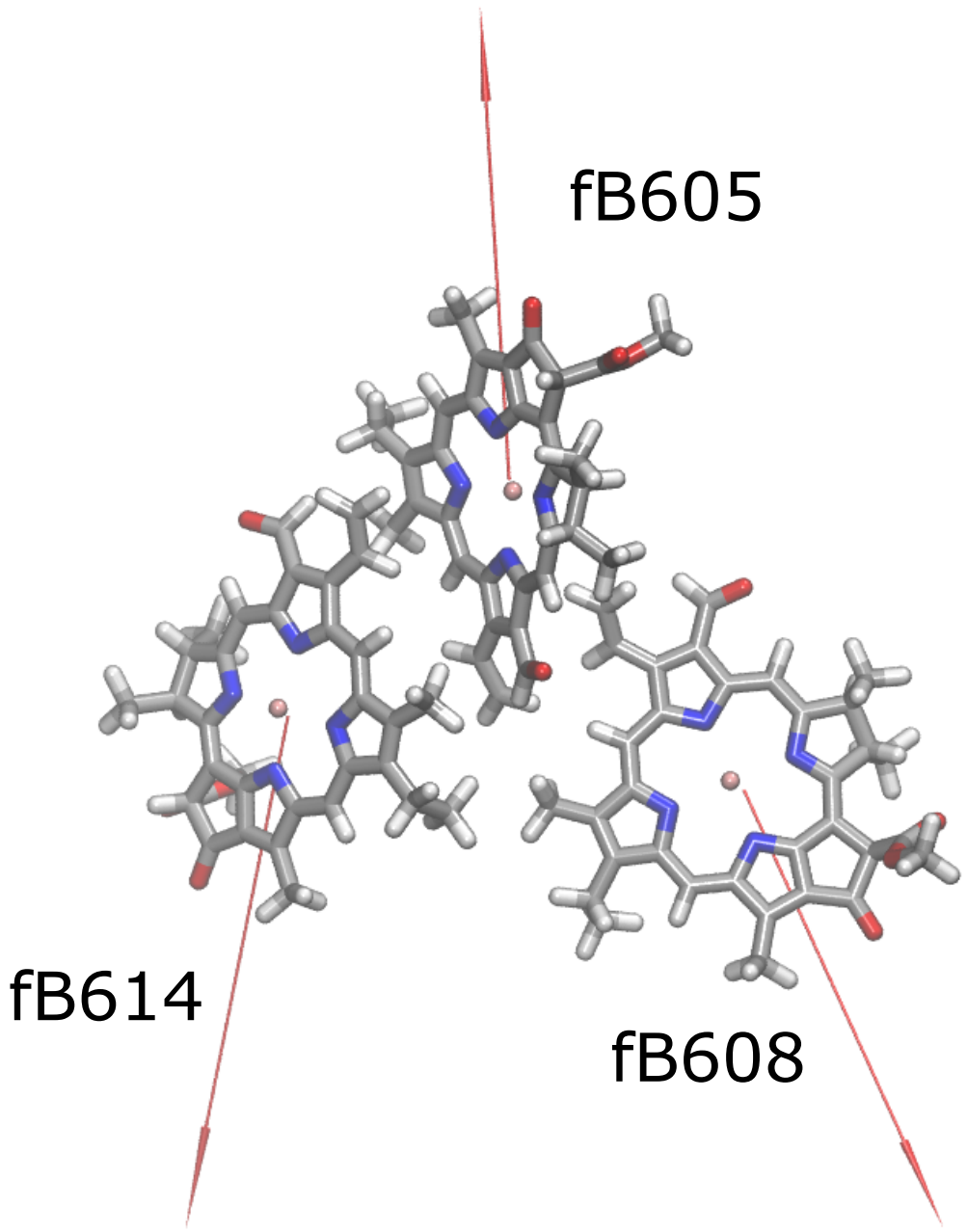


**Figure S12:** Representation of the transition dipole arrangement of the Chls in CP47 according to our calculations.

**Table S7:** Original couplings $V_{ij}$ and sign-corrected couplings $\tilde{V}_{ij} = \mathrm{sgn}(\mathbf{d}_i \cdot \mathbf{d}_j)V_{ij}$ for the most strongly coupled Chls in CP47, using the $\omega$B97X-D* functional. The sign of $\tilde{V}_{ij}$ is positive when the upper exciton is brighter than the lower exciton, and negative otherwise.

| Pair | $V_{ij}$ | $\tilde{V}_{ij}$ |
|---|---|---|
| $f$B605–$f$B608 | 115.5 | -115.5 |
| $f$B605–$f$B614 | -50.1 | 50.1 |
| $f$B608–$f$B614 | 58.6 | 58.6 |

## S9 Additional Data for the Absorption Spectra

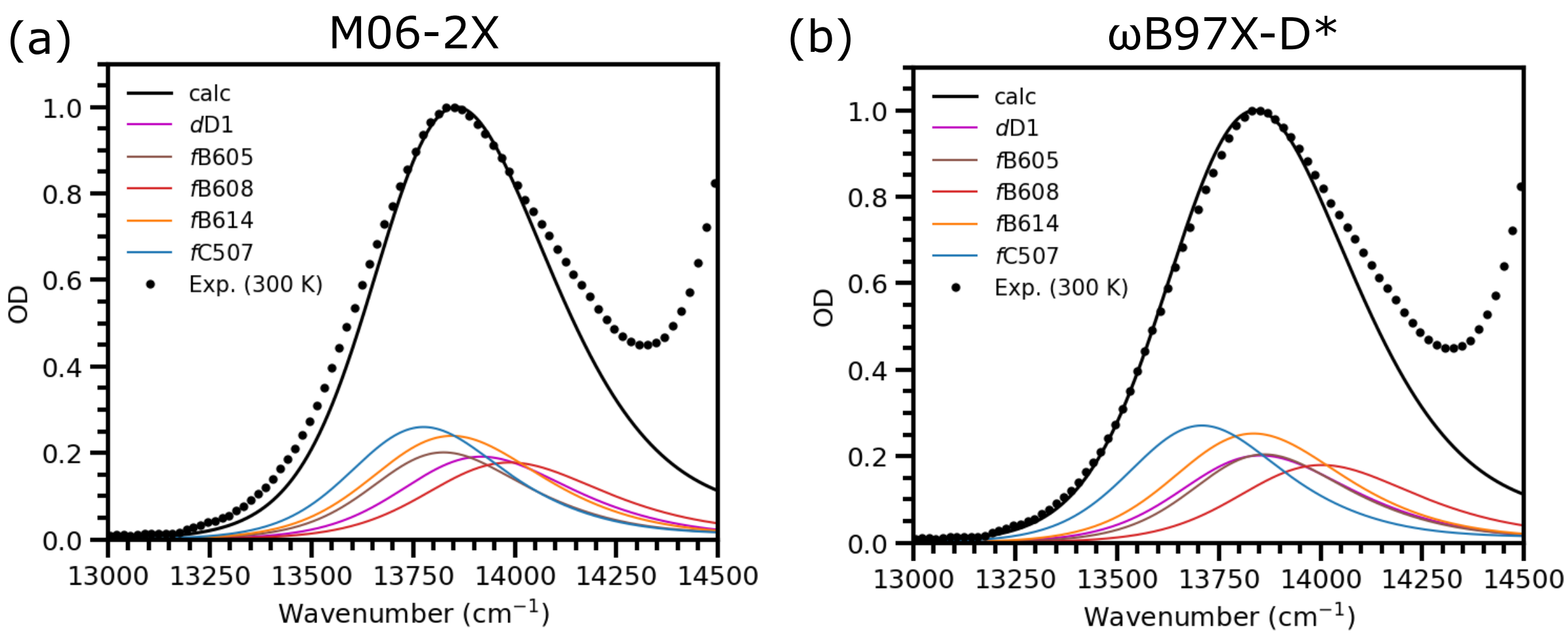


**Figure S13:** Calculated absorption spectra of the Chl *f* aggregate using the full cumulant expansion and using the excitonic Hamiltonian constructed with site energies and considering zero electronic couplings. The spectra are computed at (a) M06-2X and (b) $\omega$B97X-D* levels of theory. The black solid line shows the total calculated spectrum, while the black dots correspond to the experimental absorption spectrum at 300 K taken from Ref.[S4] Colored curves represent the site-projected contributions of the individual Chl *f* pigments (*d*D1, fB605, fB608, fB614, and fC507) to the total spectrum. The spectra obtained with TDDFT were globally shifted by 1800 cm$^{-1}$ and 930 cm$^{-1}$, for M06-2X and $\omega$B97X-D* functionals, respectively, in order to match the highest experimental peak.

**Table S8:** Comparison between the experimental exciton energies extracted from the 2DES measurements of the far-red PSII complex and the exciton energies calculated with the $\omega$B97X-D* functional. The last column reports the assignment of the corresponding exciton state obtained from our calculations.

| Exp (nm) | Exp ($cm^{-1}$) | $\omega$B97X-D* ($cm^{-1}$)[b] | Assignment |
|---|---|---|---|
| 738 | 13550 | 13864 (10.6) | fC507 |
| 734 | 13624 | 13940 (1.1) | exc fB614 |
| 727 | 13755 | 14020 (8.0) | dD1 |
| 725 | 13793 | 14089 (24.4) | exc fB605 |
| 715 | 13986 | 14329 (3.1) | exc fB608 |

[a] Experimental energies extracted from the analysis of the 2DES measurements reported in Ref.[S5]
[b] The computed energies are shifted by a constant of 930 $cm^{-1}$ as discussed in the main text. The squared transition dipoles are given in parentheses.

**Table S9:** Comparison between the experimental exciton energies extracted from the 2DES measurements of the far-red PSII complex and the exciton energies calculated with the M06-2X functional. The last column reports the assignment of the corresponding exciton state obtained from our calculations.

| Exp (nm) | Exp ($cm^{-1}$) | M06-2X ($cm^{-1}$)[b] | Assignment |
|---|---|---|---|
| 738 | 13550 | 13919 (1.6) | fC507 |
| 734 | 13624 | 13931 (11.1) | exc fB614 |
| 727 | 13755 | 14072 (20.0) | exc fB605 |
| 725 | 13793 | 14086 (14.0) | dD1 |
| 715 | 13986 | 14324 (3.3) | exc fB608 |

[a] Experimental energies extracted from the analysis of the 2DES measurements reported in Ref.[S5]
[b] The computed energies are shifted by a constant of 1800 $cm^{-1}$ as discussed in the main text. The squared transition dipoles are given in parentheses.

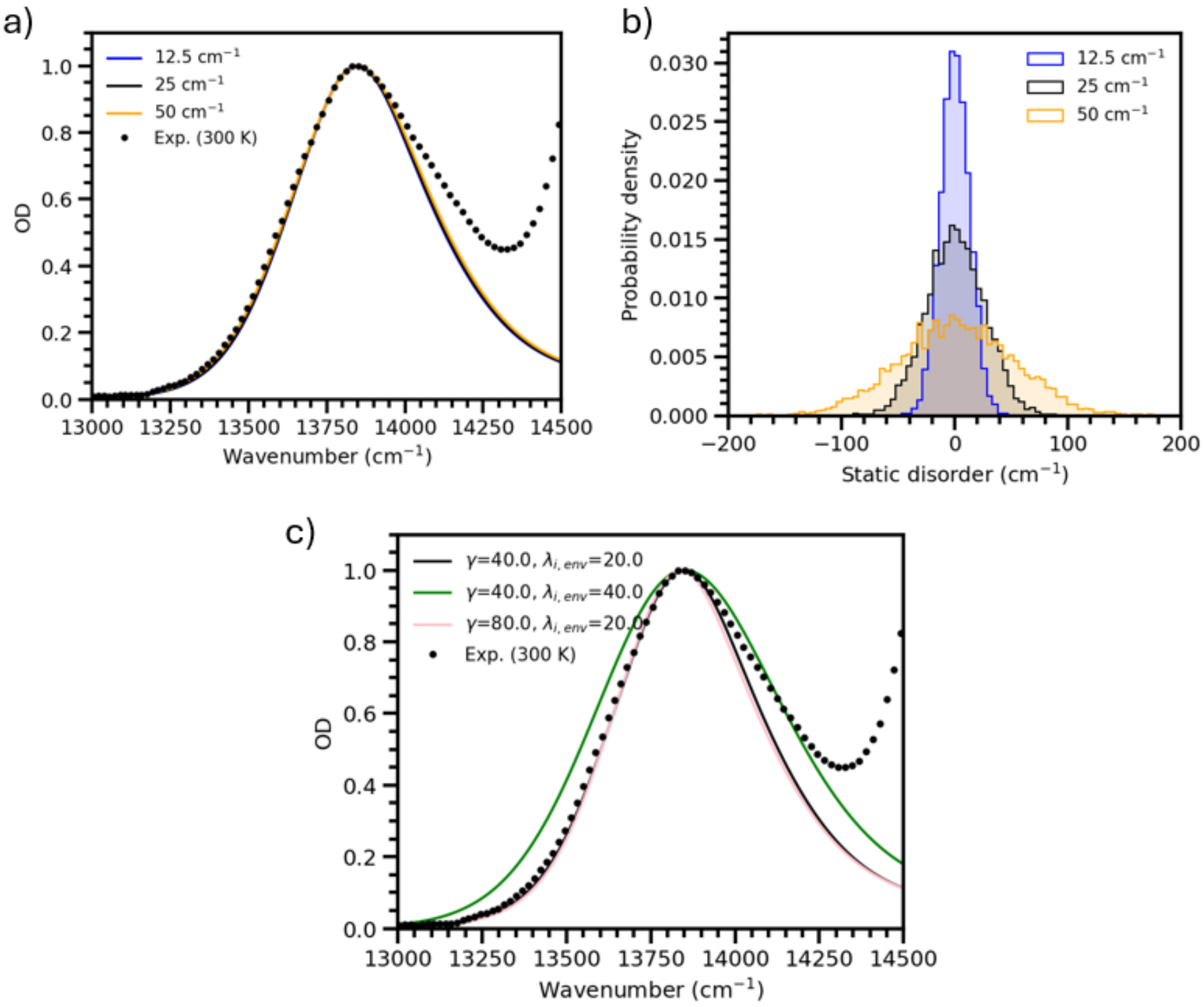


**Figure S14:** Sensitivity of the calculated absorption spectrum computed at $\omega$B97X-D* levels of theory, to the parameters describing static and dynamic environmental disorder. (a) Normalized absorption spectra calculated at 300 K using Gaussian static disorder with $\sigma$ = 12.5, 25, and 50 cm$^{-1}$, compared with the experimental spectrum. (b) Corresponding Gaussian distributions of the site-energy fluctuations. (c) Normalized absorption spectra obtained using different parameters for the environmental Brownian-oscillator contribution to the spectral density: the reference values $\gamma = 40$ cm$^{-1}$ and $\lambda_{\mathrm{i,env}} = 20$ cm$^{-1}$, a doubled reorganization energy $\lambda_{\mathrm{i,env}} = 40$ cm$^{-1}$, and a doubled damping parameter $\gamma = 80$ cm$^{-1}$. When one parameter is varied, the other is kept at its reference value. Experimental data were measured at 300 K.

# S10 Energy Transfer Rates

Excitation energy transfer rates were calculated using both Förster theory and the combined Redfield–Förster approach. We refer to Ref. S1 for a detailed explanation about the theory. In brief, in Förster theory, each pigment is treated as a localized donor or acceptor, and transfer is described perturbatively through the electronic coupling and the spectral overlap between donor emission and acceptor absorption. The Redfield–Förster approach accounts additionally for excitonic delocalization within groups of strongly coupled pigments: relaxation between exciton states belonging to the same domain is treated using secular Redfield theory, whereas transfer between different excitonic domains is described using generalized Förster theory. Thus, the latter approach is more appropriate when some antenna pigments cannot be regarded as independent localized donors. The simulation of EET dynamics have been performed with the pyQME software. The version employed for this work can be found at Zenodo https://doi.org/10.5281/zenodo.15775620.

We calculated EET rates from antenna Chls $f$ to Chl $d$D1 based on the $\omega$B97X-D* Hamiltonian, and using the same disorder parameters and spectral densities as used to produce the spectra in Fig. 6b of the main text. For the generalized Förster theory formalism, the three CP47 Chls $f$ were considered as an excitonic domain. In both generalized Förster and standard Förster cases, we calculated the effective rate from CP47 to Chl $d$D1 as an average over the donor states weighted by the equilibrium populations within the CP47 domain. The Chl $f$C507 $\rightarrow$ Chl $d$D1 rate was calculated with Förster theory.

We report in Fig. S15 the distribution of EET rates calculated with generalized Förster. The Chl $f$C507 $\rightarrow$ Chl $d$D1 EET rate is about (60 ps)$^{-1}$, and faster than the (370 ps)$^{-1}$ rate from the CP47 domain. The latter becomes slightly faster if one neglects exciton delocalization effects, *i.e.* using standard Förster theory. This effect arises from the sub-radiant nature of the lowest exciton in the CP47 cluster, which reduces the effective coupling with the RC Chl $d$D1.

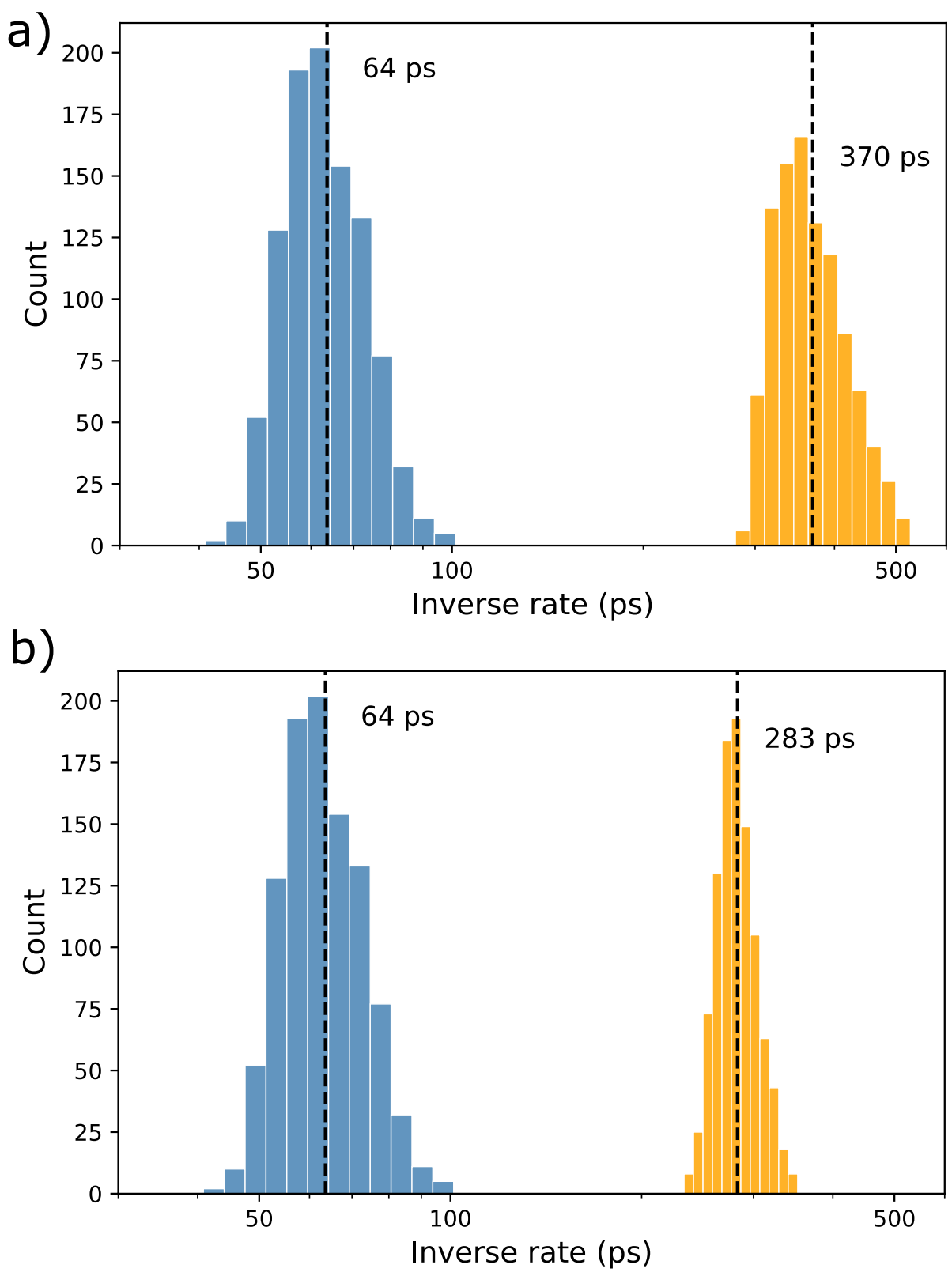


**Figure S15:** Distributions of the inverse EET rates (*i.e.* characteristic transfer times) from CP43 (blue) and CP47 (orange) to Chl dD1, calculated using (a) the combined Redfield–Förster approach and (b) Förster theory. Dashed lines indicate the corresponding characteristic average transfer times. Note the log-scale on the x-axis.

# References

[S1] Saraceno, P.; Sláma, V.; Cupellini, L. First-principles simulation of excitation energy transfer and transient absorption spectroscopy in the CP29 light-harvesting complex. *J. Chem. Phys.* **2023**, *159*, 184112.

[S2] Mukamel, S. *Principles of Nonlinear Optical Spectroscopy*; Oxford Series in Optical and Imaging Sciences; Oxford University Press: New York, 1995; Vol. 6; p 543.

[S3] Cignoni, E.; Cupellini, L.; Mennucci, B. Machine Learning Exciton Hamiltonians in Light-Harvesting Complexes. *J. Chem. Theory Comput.* **2023**, *19*, 965–977.

[S4] Gisriel, C. J.; Shen, G.; Ho, M.-y.; Kurashov, V.; Flesher, D. A.; Wang, J.; Armstrong, W. H.; Golbeck, J. H.; Gunner, M. R.; Vinyard, D. J.; Debus, R. J.; Brudvig, G. W.; Bryant, D. A. Structure of a monomeric photosystem II core complex from a cyanobacterium acclimated to far-red light reveals the functions of chlorophylls d and f. *J. Biol. Chem.* **2022**, *298*, 101424.

[S5] Silori, Y.; Willow, R.; Nguyen, H. H.; Shen, G.; Song, Y.; Gisriel, C. J.; Brudvig, G. W.; Bryant, D. A.; Ogilvie, J. P. Two-Dimensional Electronic Spectroscopy of the Far-Red-Light Photosystem II Reaction Center. *J. Phys. Chem. Lett.* **2023**, *14*, 10300–10308.
